# Deconvoluting the Photonic and Electronic Response of 2D Materials: The Case of MoS_2_

**DOI:** 10.1038/s41598-017-16970-6

**Published:** 2017-12-05

**Authors:** Kehao Zhang, Nicholas J. Borys, Brian M. Bersch, Ganesh R. Bhimanapati, Ke Xu, Baoming Wang, Ke Wang, Michael Labella, Teague A. Williams, Md Amanul. Haque, Edward S. Barnard, Susan Fullerton-Shirey, P. James Schuck, Joshua A. Robinson

**Affiliations:** 1 Department of Materials Science and Engineering, Center for 2-Dimensional and Layered Materials, The Pennsylvania State University, University Park, Pennsylvania, 16802 United States; 2 Center for Atomically Thin Multifunctional Coatings (ATOMIC), The Pennsylvania State University, University Park, Pennsylvania, 16802 United States; 3 The Molecular Foundry, Lawrence Berkeley National Laboratory, Berkeley, California 94720 United States; 4 Department of Electrical and Computer Engineering, University of Pittsburgh, Pittsburgh, Pennsylvania 15213 United States; 5 Department of Chemical and Petroleum Engineering, University of Pittsburgh, Pittsburgh, Pennsylvania 15213 United States; 6 Department of Mechanical & Nuclear Engineering, The Pennsylvania State University, University Park, Pennsylvania, 16802 United States; 7 Materials Characterization Laboratory, Materials Research Institute, The Pennsylvania State University, University Park, Pennsylvania, 16802 United States; 8 Nanofabrication Laboratory, Materials Research Institute, The Pennsylvania State University, University Park, Pennsylvania, 16802 United States

## Abstract

Evaluating and tuning the properties of two-dimensional (2D) materials is a major focus of advancing 2D science and technology. While many claim that the photonic properties of a 2D layer provide evidence that the material is “high quality”, this may not be true for electronic performance. In this work, we deconvolute the photonic and electronic response of synthetic monolayer molybdenum disulfide. We demonstrate that enhanced photoluminescence can be robustly engineered via the proper choice of substrate, where growth of MoS_2_ on r-plane sapphire can yield >100x enhancement in PL and carrier lifetime due to increased molybdenum-oxygen bonding compared to that of traditionally grown MoS_2_ on c-plane sapphire. These dramatic enhancements in optical properties are similar to those of super-acid treated MoS_2_, and suggest that the electronic properties of the MoS_2_ are also superior. However, a direct comparison of the charge transport properties indicates that the enhanced PL due to increased Mo-O bonding leads to p-type compensation doping, and is accompanied by a 2x degradation in transport properties compared to MoS_2_ grown on c-plane sapphire. This work provides a foundation for understanding the link between photonic and electronic performance of 2D semiconducting layers, and demonstrates that they are not always correlated.

## Introduction

Transition metal dichalcogenides (TMDs) exhibit promising electronic^1,2^, optoelectronic^3–6^ and piezoelectric^7^ properties with tunable band gaps^8–11^. In monolayer form, semiconducting TMDs exhibit a direct band gap^9^ and high on-off ratio^12^. Powder vaporization (PV, known as chemical vapor deposition, CVD, in other previous studies)^13–15^ is perhaps the most popular synthesis technique because of the high growth rates and ability to produce large domains^13,14,16^. However, PV-grown MoS_2_ can exhibit a wide variety of defects, each of which have been predicted to impact the optoelectronic properties of the monolayers^17,18^. The electronic performance (mobility, on/off ratio and subthreshold swing) can provide critical information on the transport properties of these monolayer MoS_2_ films and the impact of defects, but this often involves significant efforts in device fabrication that lead to transistor performance far below the predicted values^19,20^. Furthermore, the performance can vary by orders of magnitude based on the choice of substrates^14,21^ and nature/quality of electrical contacts^22^. As a result, many have turned to photoluminescence (PL) as a means to verify the quality of synthetic MoS_2_, which has driven PL enhancement as one approach to improving MoS_2_ quality^23–25^. However, PL may not be the most reliable indicator of the film quality due to the presence and potentially counter-acting effects of grain boundaries^13,14^, defects^25,26^, charge puddles^4,27^, and the supporting nanostructures^28^. Therefore, it is necessary to directly correlate structural properties and the defect-related excitonic dynamics to the electronic performance of synthetic 2D materials.

Here, we compare the photonic properties of grain boundaries between aligned and misaligned MoS_2_ monolayers. While the boundaries are reported to have little effect on the electronic properties^13,29^, we demonstrate that misaligned MoS_2_ domain boundaries exhibit a dramatic enhancement (~7×) in the PL and longer excited-state lifetimes compared to boundaries between aligned domains. This is due to high density of vacancies at misaligned boundaries that leads to significant molybdenum-oxygen (Mo-O) bonding in these locations. Subsequently, we demonstrate that this type of bonding can be engineered during MoS_2_ synthesis via proper substrate choice. While we achieve a global enhancement in PL by >100× with an r-plane sapphire substrate, electrical measurements of field effect transistors (FET) reveal a simultaneous 2–3× degradation in field-effect mobility, contact resistance, and sheet resistance. This demonstrates that the enhancement in photonic properties and electronic properties of 2D materials are not always directly correlated, and that careful control of defects and 2D/substrate interaction is critical for realizing high quality optoelectronic 2D layers.

## Results and Discussion

**Figure 1:**
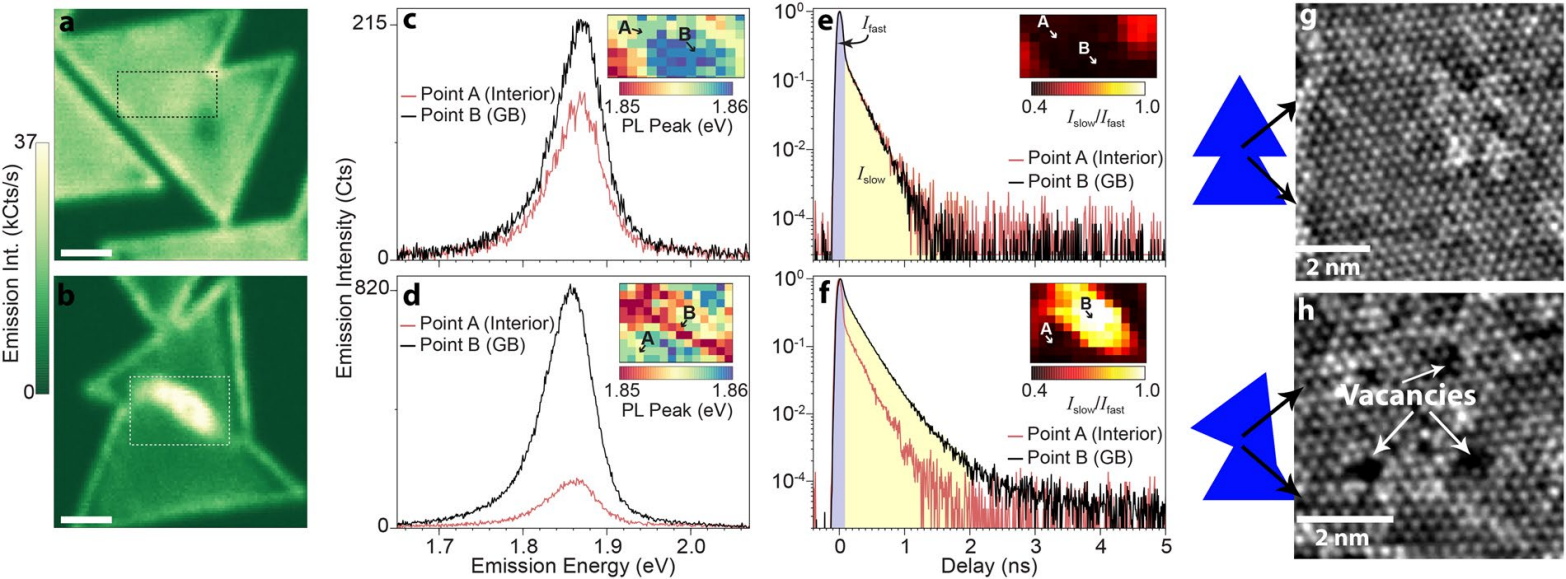
Local PL enhancement and non-radiative rate suppression mediated by defects in monolayer MoS_2_. µPL imaging of excitonic emission from (**a**) aligned and (**b**) misaligned grain boundaries. Scale bar: 2 μm Comparison of emission spectra collected from diffraction-limited regions of domain centers (red curve) and grain boundary regions (black curve) for (**c**) aligned and (**d**) misaligned grain boundary regions. Insets in (**c**) and (**d**) are spatial maps of the local peak positions of the PL from the regions denoted in panels (**a**) and (**b**), respectively. Comparison of the excited state relaxation dynamics collected from diffraction-limited regions of domain centers (red curve) and grain boundary regions (black curve) for (**e**) aligned and (**f**) misaligned grain boundary regions. In both cases, the decay transients of the domain centers are resolution limited and the decay transients reflect the instrument response of the system. The total intensity of the decay transients is divided into fast (0–100 ps) and slow (>100 ps) components, where the insets in (**e**) and (**f**) show the ratio of the fast and slow components mapped out using hyperspectral µTRPL imaging of the regions denoted in panels (**a**) and (**b**), respectively. HR-STEM of grain boundaries formed when (**g**) aligned and (**h**) misaligned domains merge during growth.

The degree of MoS_2_ domain alignment profoundly impacts the PL and excited state dynamics in the vicinity of the grain boundary. When aligned (Fig. 1(a,c)), the spatial distribution of the PL intensity at the domain boundary is nearly indistinguishable from that of the domain interior (i.e. a boundary is not readily apparent in the spatial distribution of the PL). In contrast, when the crystalline domains are not aligned (Fig. 1(b,d)), the PL intensity is significantly enhanced (~700%) near the grain boundary. It is noticed that the domain edges also exhibit PL enhancement, which can be due to the edge dipoles and impurities observed previously^30^. Additionally, micro time-resolved photoluminescence (μTRPL) of the domain boundaries (Fig. 1(e,f)) indicates an extended excited state lifetime along the grain-boundary region when domains are misaligned. Aligned domain boundaries (Fig. 1(e)) exhibit PL transients that are resolution-limited, indicating that the excited state lifetimes in both regions are less than 20 ps. In contrast, the excited state lifetime is significantly lengthened at the boundary when the domains are not aligned (Fig. 1(f)). In this region, the excited state lifetime is extended to ~150 ps. Dividing the µTRPL transients into fast (*I*_fast_; 0–100 ps) and slow (*I*_slow_; 100–5000 ps) components and plotting their ratio (*I*_slow_/*I*_fast_) provides a simple metric to quantify the lifetime increase. Evident from Fig. 1b and f (inset), the lifetime enhancement occurs over a broad region surrounding the misaligned boundary, indicating enhancement is well beyond the nanometer length scales of the boundary itself.

Photoluminescence and exciton carrier lifetime enhancement in MoS_2_ is the result of defects. This is evident when evaluating the domain boundaries via high-resolution scanning transmission electron microscopy (HR-STEM). Aligned domains (Fig. 1(g)) appear to exhibit well-matched lateral atomic arrangements that do not appear to form a boundary when they merge during growth. In comparison, randomly oriented domains (Fig. 1(h)) are not atomically matched when they merge, resulting in a high density of Mo vacancies along a clear grain boundary that separates the two misaligned domains (Figure S1)^17^. Thus, HR-STEM, combined with PL mapping, strongly indicates that it is the presence of defective regions near the boundaries of misaligned domains that are responsible for the substantially brighter PL emission in these regions. This counterintuitive trend, where a more defective material yields stronger PL, has been attributed to local charge transfer between absorbed oxygen and Mo vacancies which deplete the electron concentration of the typically n-doped MoS_2_^24,25^. Such defect-mediated compensation doping reduces the formation rate of trions, yielding a larger population of neutral excitons with higher PL quantum yield^24,31^. However, if the enhancement were due solely to a transition from trions to excitons, the emission spectrum would exhibit a sizable shift (~30 meV) to higher energies^31^, but as seen in the emission spectra, the brighter gain boundary is lower in energy than the dimmer interior. One possibility is that decreased tensile strain near the grain boundaries leads to higher exciton energies. But micro-Raman characterization of the domains and domain boundaries (Figures S2 and S3) indicates there are no discernable strain effect within the vicinity of the grain boundary regions. Furthermore, the µTRPL spectroscopy reveals an enhancement of excited state lifetime (from <20 ps to ~150 ps) at the domain boundaries that is significantly larger than what is theoretically predicted for thermalized populations of trions and excitons^32^ at 300 K. While higher-resolution lifetime and Raman studies are needed to explore this enhancement at smaller length scales, it appears that in addition to a charge-transfer mechanism, suppression of non-radiative relaxation processes for the exciton states may also play a role.

To minimize such anisotropic photonic properties caused by grain boundaries, epitaxial growth on crystalline substrates^29^ is important. Correlating the substrate surface properties and domain alignment percentage (Figs S4–S9) strongly suggests that the surface energy of the sapphire substrate prior to synthesis controls the interaction between the MoS_2_ and sapphire, thereby dominating the ability to achieve epitaxy of MoS_2_ on Al_2_O_3_^29,33^. A comprehensive evaluation of substrate annealing conditions (Table S1) indicates that the ideal treatment of 1150 °C for 8 hr in an air ambient prior to MoS_2_ growth orients significant fractions of the MoS_2_ domains to be properly oriented such that domain boundaries are minimized.

The enhanced quantum yield from the defects can be engineered and extended to the entire MoS_2_ monolayer by controlling the MoS_2_/substrate interface. Contrary to chemical routes^23,24^, controlling the atomic structure of the growth substrate is a robust route for achieving a high PL quantum yield. The typical c-plane oriented sapphire (c-sapphire) exhibits an aluminum (Al) surface termination^34,35^, which can be tuned between Al and a mixed Al-O termination at the steps (Fig. S10). This alteration of surface terminations leads to substantially different charge-transfer between the sapphire and MoS_2_. The Al terminated surface induces free-electron transfer into the MoS_2_ film due to the unsatisfied valence orbital of the top layer of Al, whereas the presence of Al-O termination reduces the electron doping to the MoS_2_ film due to the presence of Al-O bonds^35^. The reduction in free-electron transfer is likely responsible for the enhanced PL and carrier lifetimes noted at domain boundaries, and therefore, engineering this property may be desirable. The fraction of O termination can be further increased by considering different orientations of sapphire, such as r-plane sapphire, which is typically oxygen terminated^36^ and can be protonated due to the presence of water in the air^37^. In our case, MoS_2_ is grown at 800 °C, which likely leads to deprotonation and a surface that is O-terminated^38^. As a result, there is a direct interaction between an O-terminated sapphire surface rather than an Al-terminated surface (in the case of c-plane sapphire), and the free-electron transfer from sapphire to MoS_2_ is minimized.

**Figure 2:**
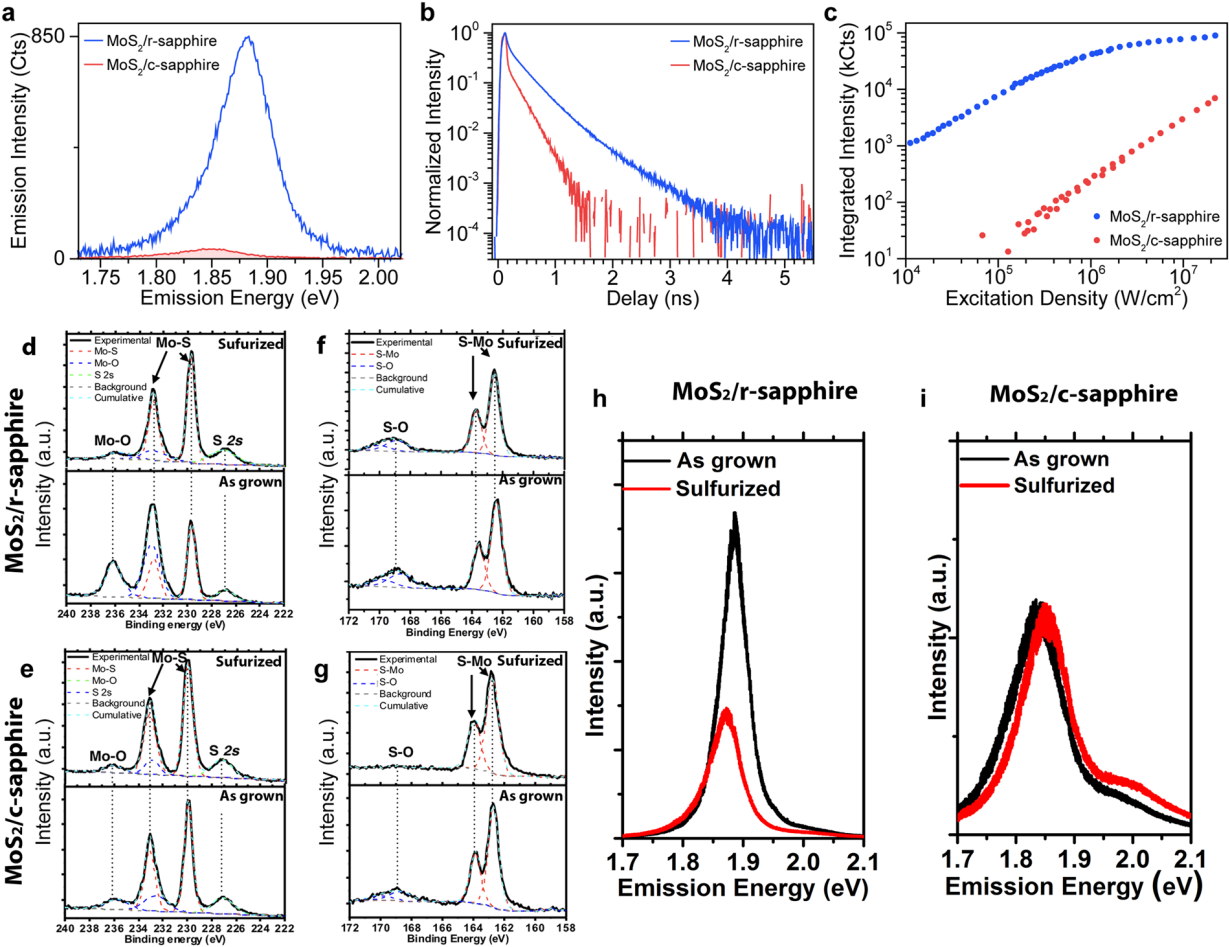
Photoluminescence enhancement and non-radiative rate suppression by oxygen bonding in monolayer MoS_2_ grown on r-sapphire. (**a**) Comparison of the PL spectra of monolayer MoS_2_ grown on c-sapphire (MoS_2_/c-sapphire; red curve) to that of monolayer MoS_2_ grown on r-sapphire (MoS_2_/r-sapphire; blue curve) reveals that the PL of MoS_2_/r-sapphire is substantially brighter and shifted to higher energies. (**b**) Likewise, the excited state lifetime of MoS_2_/r-sapphire (red curve) is clearly enhanced compared that of MoS_2_/c-sapphire. (**c**) As a direct result of the longer excited state lifetimes, the scaling of the emission intensity of the PL with excitation density of MoS_2_/r-sapphire (blue curve) shows an earlier onset of exciton-exciton annihilation (i.e., sublinear behavior) than that of MoS_2_/c-sapphire which is linear over the same range of excitation densities and ~100× dimmer. (**d–e**) The XPS spectra in the Mo range of MoS_2_ on r-sapphire (**d**) and c-sapphire (**e**). The Mo-O bonding is also clearly identified in Mo 3d range when the growth is on r-sapphire (**d**) but negligible on c-sapphire. (**e**) The reduced oxygen concentration agrees well with the quenched PL intensity in Fig. 3h, indicating the oxygen doping is the key factor of the PL enhancement on r-sapphire. (**f–g**) XPS spectra in the S range of MoS_2_ on r-sapphire (**f**) and c-sapphire (**g**). In addition to the expected S 2p peak, S-O bonding is observed in both as-grown samples. After the sulfurization, the S-O bonding can be reduced on c-sapphire, indicating the S-O in this case is due to the unsulfurized MoO_x_. (**h**) PL spectra of MoS_2_/r-sapphire before and after sulfurization. The PL quenches ~51% after the sulfurization. (**i**) PL spectra of MoS_2_/c-sapphire before and after sulfurization. The PL is ~10 meV blue shifted after the sulfurization, which may be due to the reduction of S vacancies after the sulfurization.

Use of r-plane sapphire leads to dramatic enhancements in the photophysical properties of monolayer MoS_2_. This is immediately evident when considering the PL of MoS_2_ on r-sapphire (Fig. 2a), which is significantly brighter (100×) and shifted by ~30 meV to higher energies (Fig. S11) when compared to traditional c-plane sapphire. The spectral shift is consistent with a lower density of trions due to a reduction in the electron density in the MoS_2_ on the r-plane sapphire^31,39^. A significant difference is also observed in the excited state lifetimes of the photoexcitations (Fig. 2(b)), where r-plane sapphire yields a MoS_2_ excited state lifetime increase of >30× to approximately 500 ps. Such an apparent reduction of relaxation rates significantly impacts the exciton dynamics in the system, similar to what was observed at the misaligned grain boundaries. Figure 2(c) compares the PL emission intensity as a function of excitation density between MoS_2_ on c-sapphire to MoS_2_ on r-sapphire. MoS_2_ on c-sapphire maintains a linear dependence across the full excitation range. However, MoS_2_ on r-sapphire transitions from a linear to sublinear dependence at an excitation density of ~10^5^ W/cm^2^. This threshold marks the critical exciton density (i.e., the number of excitons injected into the MoS_2_ via pulsed excitation) where exciton-exciton annihilation processes begin to dominate the relaxation dynamics^40^. This excitation density depends on the overall relaxation rate, and its lower value for the r-sapphire results from the longer excited state lifetimes^40^ (see Figure S12 and accompanying discussion). Such a phenomenon provides evidence that the substrate provides a route to control the photophysical dynamics of monolayer MoS_2_.

The enhanced photoluminescence and excited state lifetime of monolayer MoS_2_ on r-sapphire is due to Mo-O bonding potentially at the MoS_2_/sapphire interface. Because the reduction of non-radiative recombination on the r-plane sapphire is not spatially isolated to grain boundaries or edges and is wide-spread across the sample, x-ray photoelectron spectroscopy (XPS) can provide information on the compositional differences of the MoS_2_/substrate interface on r- and c-sapphire (Fig. 2(d–g)) that give rise to the differences in the photodynamics. Beyond the dominant Mo 3d and S 2p peaks^15,41^, we identify peaks at 168.9 eV and 236.2 eV for both substrate configurations (Fig. 2(d–g)), corresponding to S-O and Mo-O bonding, respectively^25,41,42^. Compared to MoS_2_ on c-sapphire, the higher Mo-O peak intensity for MoS_2_ on r-sapphire (Fig. 2(d, e)) indicates a higher concentration of Mo-O bonding in MoS_2_ when grown on r-sapphire. To further understand the role of the S-O and Mo-O bonding on the optical properties, we thermally treat the samples in a sulfur-rich environment (sulfurization) and re-evaluate the chemical bonding by XPS (Fig. 2(d–g)). Following sulfurization, the S-O bond in MoS_2_ on c-sapphire is eliminated (Fig. 2(f,g)), but persists in the MoS_2_ on r-sapphire, suggesting that the S-O bond is fundamentally different between the two growth substrates. The S-O bond on c-sapphire is likely due to unsulfurized MoO_x_S_2−x_, while the S-O bond on r-sapphire is more likely to be from S bonded with the top O-terminated sapphire due to the weak O-O bond in sapphire^43^ and strong interfacial interaction when O is terminated on the r-sapphire surface^44^. Additionally, the Mo-O bonding on r-sapphire is significantly reduced (Fig. 2(d,e) sulfurized) and the PL is quenched by ~51% intensity (area under the curve) and ~17 meV red shift after sulfurization (Fig. 2(h,i)), while MoS_2_ on c-sapphire exhibits nearly no change in the Mo-O peak or PL quench after such treatment. Therefore, the presence of the Mo-O bonding, which is much more significant with r-sapphire, can be considered a dominating factor in the PL enhancement of monolayer MoS_2_.

**Figure 3:**
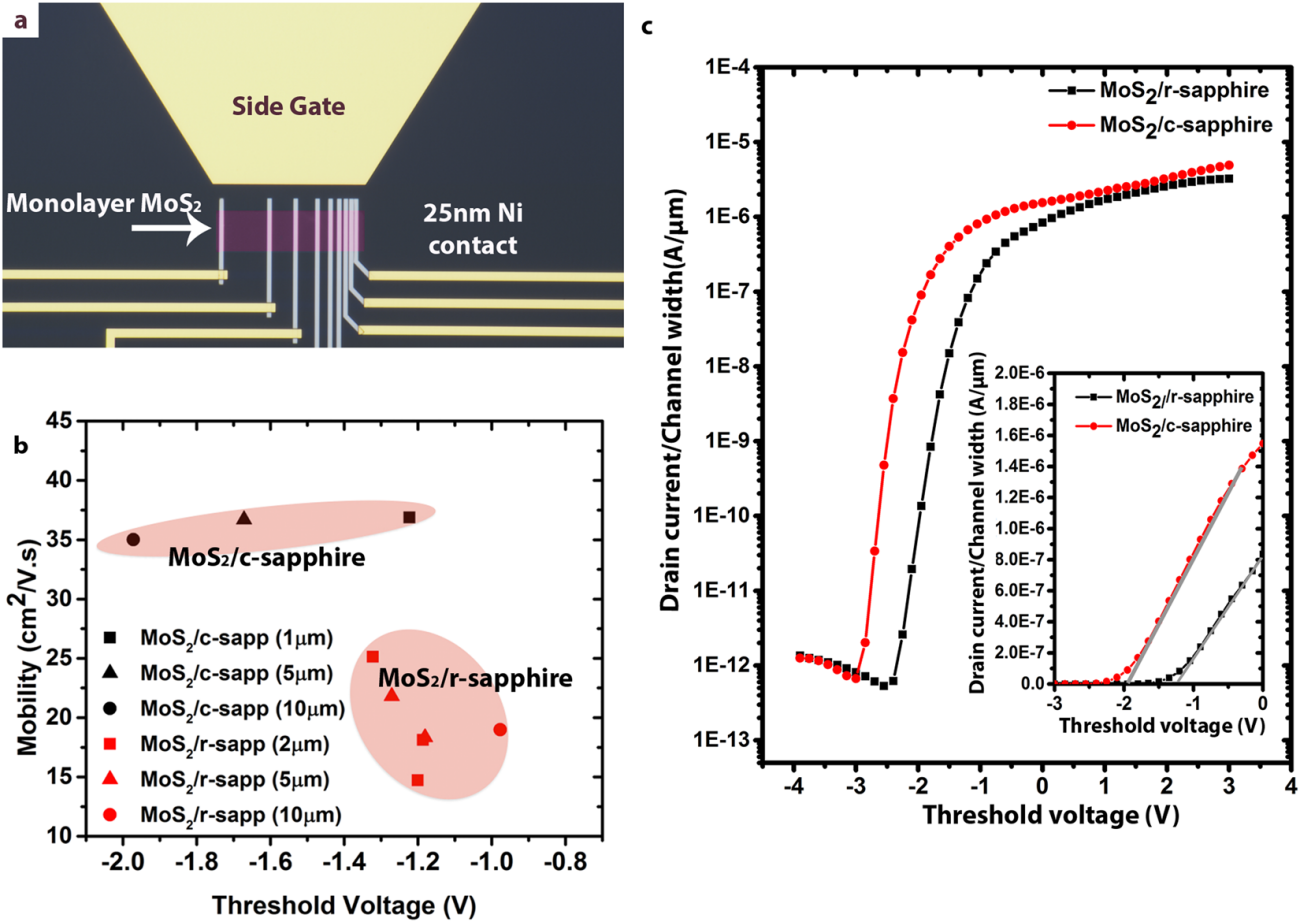
FET device comparison for monolayer MoS_2_/r-sapphire and MoS_2_/c-sapphire. **(a**) 100× optical microscopy (OM) image of a TLM device, which consists of back-back FETs of varying channel length. Note that the MoS_2_ channel is false-colored for easier visualization. The side-gate structure at the top of the image is used to make contact with the gate probe in order to ensure constant side-gate-MoS_2_ distance and consistent, efficient ion response across multiple devices and device structures. (**b**) A plot of field-effect mobility vs threshold voltage for the devices on the two different sapphire surfaces. MoS_2_/r-sapphire devices are clearly p-doped relative to MoS_2_/c-sapphire and also suffer from slightly lower mobility which is attributed to increased carrier scattering from O-defects in the film as a result of the r-plane surface termination. (**c**) Comparison of transfer curves between identical MoS_2_/r-sapphire and MoS_2_/c-sapphire devices, further demonstrating the threshold voltage shift for the different surface terminations as well as the high on/off ratio and steep turn-on for both cases. In this instance, L_ch_ = 10 µm, drain current is normalized by channel width, and V_d_ = 500 mV. The inset of (**c**) is a magnified plot of the same curves in linear scale to better visualize the actual threshold voltage positions and difference in transconductance (g_m_) represented by the slope.

Although the Mo-O bonding leads to significant enhancements in photonic properties, this does not mean the electronic properties are also improved. To evaluate how photonic and electronic properties are linked, we fabricate MoS_2_ field effect transistors (FETs) on c-plane and r-plane sapphire, and evaluate the transport properties using a solid-polymer electrolyte gate: polyethylene oxide (PEO)-CsClO_4_ (See SI for experimental details)^45^. An optical micrograph of a device prior to electrolyte deposition is shown in Fig. 3(a). Evident in Fig. 3b, monolayer MoS_2_ on c-sapphire exhibits improved transport properties compared with r-sapphire, as shown in Fig. 3b and c. MoS_2_/r-sapphire devices exhibit reduced electronic performance, specifically a field-effect mobility (µ_FE_) ~50% lower than c-sapphire (μ_c_ = 36.2 ± 1.0 cm^2^/Vs; μ_r_ = 19.5 ± 3.6 cm^2^/Vs), 1.46x higher sub-threshold swing (SS) (SS_c_ = 114 ± 15 mV/dec; SS_r_ = 166 ± 10 mV/dec), and lower current ON/OFF ratio, which could be due to additional interaction at the MoS_2_/r-sapphire interface such as Mo-O bonding^46^. To the best of our knowledge, it also marks the highest mobility for monolayer MoS_2_ devices directly fabricated on sapphire without transfer^21^. The µ_FE_ versus threshold voltage (V_th_) is summarized in Fig. 3(b). On average, the V_th_ is positively shifted for r-plane devices compared to c-plane devices for the same channel length, reaffirming the O-mediated p-type compensation doping of MoS_2_ from Mo-O bonding predicted by theory and supported by PL measurements in this work^24,25,47^. This positive threshold voltage shift is most evident for long-channel devices where the threshold voltage difference is ~1 V (L_ch_ = 10 µm; circles in Fig. 3(b)). Representative transfer curves for L_ch_ = 10 µm devices under identical drain bias (V_d_ = 500 mV) and measurement conditions are shown in Fig. 3c where the MoS_2_/c-sapphire device displays negative shift in the V_th_ relative to gate bias (see also inset), lower SS, and higher µ_FE_ (Fig. (3c) inset). It is worth noting that the FETs transfer curves were not taken on channel lengths <1 μm due to micro-cracking, tearing and delamination during the device fabrication (Figure S13).

In addition to the FETs measurements, evaluation of the MoS_2_ properties via the transfer length method (TLM) reveals an approximate 3× higher sheet resistance (R_sh_) and contact resistance (R_c_) on MoS_2_/r-sapphire (R_sh_ = 65 kΩ/□ and R_c = _63 kΩ·µm) than MoS_2_/c-sapphire (R_sh = _29 kΩ/□ and R_c = _21 kΩ·µm) (Figure S14). The decreased mobility and increased R_sh_ for monolayer MoS_2_/r-sapphire devices can be due to increased scattering of charge carriers caused by oxygen doping and enhanced film-substrate coupling^46^. As a result, oxygen incorporation into the MoS_2_ lattice and S-O bonding with the underlying substrate, as demonstrated by XPS, is considered to disrupt the layered van der Waals nature and the periodic potential of monolayer MoS_2_, leading to a degradation in electronic properties^46,48^.

## Conclusions

This work demonstrates that optical properties may not be correlated with electronic properties in 2D materials. This is especially true in MoS_2_, where the degree of domain alignment is found to strongly influence the optical properties of the grain boundaries. In particular, grain boundaries that form when misaligned domains merge enhances the PL intensity and excited state lifetimes likely via the combined effects of defect mediated charge transfer and suppression of non-radiative recombination. This phenomenon can be directly engineered across the entire MoS_2_ layer by utilizing an O-terminated substrate surface (r-sapphire), which yields >100× increase in PL intensity and >30× increase in excited state lifetime - comparable to that reported for chemically treated MoS_2_^23^. The improved PL properties have the same characteristics as those of the grain boundaries and are attributed to changes in the inherent charge transfer between the MoS_2_ and the underlying substrate and the role of O-bonding in the enhancement. Moreover, we demonstrate that the Mo-O bonding responsible for enhanced photonic performance actually results in reduced electronic performance. The results presented here deconvolute the photonic and electronic response of monolayer MoS_2_, and show that they are not correlated.

## Methods

### MoS_2_ synthesis

Discontinuous films of monolayer MoS_2_ domains are subsequently grown by powder vaporization technique. 2 mg MoO_3_ (99.8%, Sigma Aldrich) is put in an alumina crucible located in the center of a hot wall furnace. 200 mg of S powder (99.995%, Alfa Aesar) is put in a quartz crucible located ~12 inches upstream of the MoO_3_ crucible. After 5 min of flowing 100 sccm Ar at 710 Torr, the furnace was elevated to 550 °C for 2 min as a nucleation step, followed by dwelling at 800 °C for 15 min for the growth. The sulfur powder is heated by an individual heat tape wrapped outside the furnace, and the temperature is 130 °C.

### Raman, PL and TRPL characterization

µTRPL imaging was performed on a scanning confocal microscope. Pulsed laser excitation (λ = 500 nm; 2.5 nm bandwidth; 5 ps pulse width; 40 MHz repetition rate; 500 nW CW-equivalent power) was focused onto the sample with a 100 × 0.95 NA objective to a diffraction-limited spot. Photoluminescence from the sample was collected by the same objective, and reflected laser light was filtered from the collected light using a 600 nm long-pass filter (Thorlabs) combined with a 532 nm long-pass dichroic mirror (Semrock). The filtered photoluminescence was focused onto a single-photon counting avalanche photodiode (Micro Photon Devices) where photon detection events where timed and analyzed with time-correlated single-photon counting electronics (PicoHarp). Mapping was performed by raster-scanning the sample with high precision piezo stages (Mad City Labs) and collecting a photoluminescence transient at each spatial position. The temporal resolution as estimated with the FWHM of the instrument-response function was ~50 ps. The individual spectra and photoluminescence transients, and the power-dependent intensity shown in Fig. 2 where acquired on the same microscope system. For spectral characterization, the collected light was dispersed by a spectrometer (Princeton Instruments) and detected with a cooled charge coupled device (CCD) camera (Andor). The power dependent measurements were obtained by varying the laser power and recording a full emission spectrum at each point.

Raman characterization of the aligned and misaligned grain boundaries was performed on a standard Raman microscope system (NTMDT). CW laser excitation at 532 nm with a power of 500 µW was focused by a 100× 0.6 NA objective on the sample. The same objective was used to collect the emission from the sample and then analyzed using the Raman spectroscopy system.

### HRSTEM Characterization

Aberration-corrected high-resolution scanning transmission electron microscopy (HR-STEM) images were collected using a double aberration-corrected FEI Titan3 (60–300) operating at 80 kV at Penn State. All high-resolution STEM images are captured with a high angle annular dark field (HADDF) detector. A beam current of 45 pA, beam convergence of 30 mrad, and camera length of 115 mm are used for image acquisition.

### (AR)XPS characterization

A Phi Versa Probe II is used for this analysis, with a passing energy of 23.5 eV and a step size of 0.1 eV. All of the samples are integrated at the same dwell time (200 ms) at each step. For ARXPS characterization, the characterization angle is set between 30^0^ to 85^0^ in order to probe the top 1 nm surface of the as-received sapphire and annealed sapphire substrate. The XPS data is analyzed and processed by CASA XPS software.

### Field effect transistors fabrication

An array of Ti/Au (10/90 nm) alignment/fiduciary are deposited by standard optical lithography, e-beam evaporation, and lift-off processes. Large triangular monolayer MoS_2_ flakes (40–60 μm side length) are then etched into smaller rectangular channels of varying widths and lengths (dictated by original flake size) to be used as a TLM bar of uniform composition/thickness and defined width. Following electron beam lithography (EBL), the MoS_2_ etch is carried out in a Plasma Therm PT-720 Reactive Ion Etch (RIE) tool using a gas chemistry of SF_6_/Ar/O_2_ (30/10/10 sccm) at 100 W power and 10 mTorr pressure for a total etch time of 20 seconds. 25 nm Ni source/drain extensions (grey metal fingers in Fig. 3a) are formed by EBL, e-beam evaporation, and lift-off processes. Ni is deposited at 0.5 Å/sec at a deposition pressure of 3 × 10^−6^ Torr. The width of these Ni source/drain fingers is 2 μm. Prior to Ni source/drain deposition and after e-beam resist development, samples are O_2_/He (150/50 sccm) plasma treated (50 W and 500 mTorr) for 45 seconds in an M4L etch RIE tool in order to chemically remove resist polymer residues and thereby improve the metal/MoS_2_ interface (More details about the device fabrication and discussion is in supplemental information).

### Electrolyte gate application

A solid polymer electrolyte (PEO:CsClO_4_) is used for ionic gating. The preparation of the polymer electrolyte is similar to previously published procedures^45^ with the exception that the electrolyte is prepared and deposited in an argon-filled glovebox where the concentrations of H_2_O and O_2_ are maintained to be <0.1 part-per-million (ppm). Poly(ethylene-oxide) (PEO) (molecular weight 95,000 g/mol, Polymer Standards Service) and anhydrous CsClO_4_ (99.9%, Sigma-Aldrich) are dissolved in anhydrous acetonitrile (Sigma-Aldrich) with an ether oxygen to Cs molar ratio of 76:1 to make a 1 wt% solution. The solid polymer electrolyte is deposited on the sample by drop-casting 25 µL onto the ∼1 × 1 cm^2^ sample. After a 15-minute wait time to allow the majority of the solvent to evaporate, the sample is annealed on a hotplate at 80 °C for 3 mins to drive off remaining solvent. The sample is then transferred from the glovebox to the probe station through an Ar-filled load lock. The entire process of electrolyte preparation, deposition, transfer to the probe station, and measurement are completed under an inert gas environment with no sample exposure to ambient. Electrical measurements are performed on a Lake Shore cryogenic vacuum probe station (CRX-VF) under ∼10^−6^ Torr at room temperature using a Keysight B1500A semiconductor parameter analyzer.

### Electrolyte gating measurements

All transfer curves (I_d_ − V_g_) shown are taken by sweeping V_g_ from +3 to −4 V. Prior to collecting each transfer curve, a constant gate bias of V_g_ = 3 V is applied for 5 min to allow the ions in the electrolyte/MoS_2_ system to reach equilibrium. A 5 min hold time prior to initiating transfer curve measurement is determined by monitoring I_d_ under constant gate bias (V_g_ = 3 V) and small drain bias over time; when I_d_ is stabilized, this indicates equilibrium has been established (More details and discussion is in the supplemental information).

### Data Availability Statements

All data analyzed during this study are included in this published article and its Supplementary Information files.

## Electronic supplementary material

Supplementary Information

## References

[1] Radisavljevic B, Radenovic A, Brivio J, Giacometti V, Kis A (2011). Single-layer MoS_2_ transistors. Nat. Nanotechnol..

[2] Liu W (2013). Role of metal contacts in designing high-performance monolayer n-type WSe_2_ field effect transistors. Nano Lett..

[3] Baugher BWH, Churchill HOH, Yang Y, Jarillo-Herrero P (2014). Optoelectronic devices based on electrically tunable p-n diodes in a monolayer dichalcogenide. Nat. Nanotechnol..

[4] Borys NJ (2017). Anomalous Above-Gap Photoexcitations and Optical Signatures of Localized Charge Puddles in Monolayer Molybdenum Disulfide. ACS Nano.

[5] Lopez-Sanchez O, Lembke D, Kayci M, Radenovic A, Kis A (2013). Ultrasensitive photodetectors based on monolayer MoS_2_. Nat. Nanotechnol..

[6] Wang X (2015). Ultrasensitive and Broadband MoS_2_ Photodetector Driven by Ferroelectrics. Adv. Mater..

[7] Wu W (2014). Piezoelectricity of single-atomic-layer MoS_2_ for energy conversion and piezotronics. Nature.

[8] Jariwala D, Sangwan VK, Lauhon LJ, Marks TJ, Hersam MC (2014). Emerging device applications for semiconducting two-dimensional transition metal dichalcogenides. ACS Nano.

[9] Mak KF, Lee C, Hone J, Shan J, Heinz TF (2010). Atomically thin MoS_2_: a new direct-gap semiconductor. Phys. Rev. Lett..

[10] Coehoorn R (1987). Electronic structure of MoSe_2_, MoS_2_, and WSe_2_. I. Band-structure calculations and photoelectron spectroscopy. Phys. Rev. B. Condens. Matter.

[11] Zhao W (2013). Evolution of Electronic Structure in Thin Sheets of WS_2_ and WSe_2_. ACS Nano.

[12] Fang H (2012). High-performance single layered Wse_2_ p-FETs with chemically doped contacts. Nano Lett..

[13] van der Zande AM (2013). Grains and grain boundaries in highly crystalline monolayer molybdenum disulphide. Nat. Mater..

[14] Najmaei S (2013). Vapour phase growth and grain boundary structure of molybdenum disulphide atomic layers. Nat. Mater..

[15] Lee Y-H (2012). Synthesis of large-area MoS_2_ atomic layers with chemical vapor deposition. Adv. Mater..

[16] Wu S (2013). Vapor–Solid Growth of High Optical Quality MoS_2_ Monolayers with Near-Unity Valley Polarization. ACS Nano.

[17] Zhou W (2013). Intrinsic Structural Defects in Monolayer Molybdenum Disulfide. Nano Lett..

[18] Hong J (2015). Exploring atomic defects in molybdenum disulphide monolayers. Nat. Commun..

[19] Bhimanapati GR (2015). Recent Advances in Two-Dimensional Materials Beyond Graphene. ACS Nano.

[20] Lin Z (2016). 2D materials advances: from large scale synthesis and controlled heterostructures to improved characterization techniques, defects and applications. 2D Mater..

[21] Bersch BM (2017). Selective-area growth and controlled substrate coupling of transition metal dichalcogenides. 2D Mater..

[22] Das S, Chen HY, Penumatcha AV, Appenzeller J (2013). High performance multilayer MoS2 transistors with scandium contacts. Nano Lett..

[23] Amani M (2015). Near-unity photoluminescence quantum yield in MoS_2_. Science (80-.)..

[24] Mouri S, Miyauchi Y, Matsuda K (2013). Tunable photoluminescence of monolayer MoS_2_ via chemical doping. Nano Lett..

[25] Nan H (2014). Strong photoluminescence enhancement of MoS_2_ through defect engineering and oxygen bonding. ACS Nano.

[26] Tongay S (2013). Defects activated photoluminescence in two-dimensional semiconductors: interplay between bound, charged, and free excitons. Sci. Rep..

[27] Bao W (2015). Visualizing nanoscale excitonic relaxation properties of disordered edges and grain boundaries in monolayer molybdenum disulfide. Nat Commun.

[28] Wang Z (2016). Giant photoluminescence enhancement in tungsten-diselenide–gold plasmonic hybrid structures. Nat. Commun..

[29] Dumcenco D (2015). Large-Area Epitaxial Monolayer MoS_2_. ACS Nano.

[30] Gutiérrez HR (2013). Extraordinary Room-Temperature Photoluminescence in Triangular WS_2_ Monolayers. Nano Lett..

[31] Mak KF (2012). Tightly bound trions in monolayer MoS_2_. Nat. Mater..

[32] Wang H (2016). Radiative lifetimes of excitons and trions in monolayers of the metal dichalcogenide MoS_2_. Phys. Rev. B.

[33] Ji Q (2015). Unravelling Orientation Distribution and Merging Behavior of Monolayer MoS_2_ Domains on Sapphire. Nano Lett..

[34] Wang R (2016). Atomic Step Formation on Sapphire Surface in Ultra-precision Manufacturing. Sci. Rep..

[35] Zhukovskii YF, Kotomin EA, Herschend B, Hermansson K, Jacobs PWM (2002). The adhesion properties of the Ag/α-Al_2_O_3_(0001)interface: an ab initio study. Surf. Sci..

[36] Trainor TP, Eng PJ, Brown GE, Robinson IK, De Santis M (2002). Crystal truncation rod diffraction study of the α-Al_2_O_3_ (11̄02) surface. Surf. Sci..

[37] Sung J, Zhang L, Tian C, Waychunas GA, Shen YR (2011). Surface Structure of Protonated R-Sapphire (11̄0̄2) Studied by Sum-Frequency Vibrational Spectroscopy. J. Am. Chem. Soc..

[38] Gottler B, Niemannt W, Redfern SAT (1989). EXAFS and XANES spectroscopy study of the oxidation and deprotonation of biotite. Mineral. Mag..

[39] Li Y (2014). Photoluminescence of monolayer MoS_2_ on LaAlO_3_ and SrTiO_3_ substrates. Nanoscale.

[40] Yu Y (2016). Fundamental limits of exciton-exciton annihilation for light emission in transition metal dichalcogenide monolayers. Phys. Rev. B.

[41] Addou R (2015). Impurities and Electronic Property Variations of Natural MoS_2_ Crystal Surfaces. ACS Nano.

[42] Yang Z, Dai Y, Wang S, Cheng H, Yu J (2015). *In situ* incorporation of a S, N doped carbon/sulfur composite for lithium sulfur batteries. RSC Adv..

[43] Pishchik, V., Lytvynov, L. a. & Dobrovinskaya, E. R. *Sapphire*. 10.1007/978-0-387-85695-7 (2009).

[44] Stirner T, Sun J, Aust M (2012). *Ab Initio* Hartree-Fock Simulation of R-Plane Sapphire. Phys. Procedia.

[45] Xu H, Fathipour S, Kinder EW, Seabaugh AC, Fullerton-Shirey SK (2015). Reconfigurable Ion Gating of 2H-MoTe_2_ Field-Effect Transistors Using Poly(ethylene oxide)-CsClO_4_ Solid Polymer Electrolyte. ACS Nano.

[46] Ma N, Jena D (2014). Charge Scattering and Mobility in Atomically ThinSemiconductors. Phys. Rev. X.

[47] Chakraborty B (2012). Symmetry-dependent phonon renormalization in monolayer MoS_2_ transistor. Phys. Rev. B - Condens. Matter Mater. Phys..

[48] Schmidt H (2014). Transport properties of monolayer MoS_2_ grown by chemical vapor deposition. Nano Lett..

