# Supplementary material for: Deconvoluting the Photonic and Electronic Response of 2D Materials: The Case of MoS2
Source: Sci Rep. 2017 Dec 5;7:16938. doi: 10.1038/s41598-017-16970-6 (PMC5717065; doi:10.1038/s41598-017-16970-6)
Supplement: Supplementary file 1 — Supplementary Information [file 41598_2017_16970_MOESM1_ESM.pdf]

Deconvoluting the Photonic and Electronic Response of 2D Materials: The Case of MoS_2

Kehao Zhang[1,2], Nicholas J. Borys[3 •], Brian M. Bersch[1 •], Ganesh R. Bhimanapati[1], Ke Xu[4] Baoming Wang[6], Ke Wang[7], Michael Labella[8], Teague A. Williams[1], Md Amanul. Haque[2,6], Edward S. Barnard[3], Susan Fullerton-Shirey[4,5] P. James Schuck[3], Joshua A. Robinson[1,2] [1]

1. Department of Materials Science and Engineering, Center for 2-Dimensional and Layered Materials, The Pennsylvania State University, University Park, Pennsylvania, United States, 16802
2. Center for Atomically Thin Multifunctional Coatings (ATOMIC), The Pennsylvania State University, University Park, Pennsylvania, United States, 16802,
3. The Molecular Foundry, Lawrence Berkeley National Laboratory, Berkeley California, United States, 94720
4. Department of Chemical and Petroleum Engineering, University of Pittsburgh, Pittsburgh, Pennsylvania, United States, 15213
5. Department of Electrical and Computer Engineering, University of Pittsburgh, Pittsburgh, Pennsylvania, United States, 15213
6. Department of Mechanical & Nuclear Engineering, The Pennsylvania State University, University Park, Pennsylvania, United States, 16802
7. Materials Characterization Laboratory, Materials Research Institute, The Pennsylvania State University, University Park, Pennsylvania, United States, 16802
8. Nanofabrication Laboratory, Materials Research Institute, The Pennsylvania State University, University Park, Pennsylvania, United States, 16802

- These authors contributed equally.

High resolution scanning transmission electron microscopy (HRSTEM) characterization

Aberration-corrected high-resolution scanning transmission electron microscopy (HR-STEM) images were collected using a double aberration-corrected FEI Titan3 (60-300) operating at 80 kV at Penn State. All high-resolution STEM images are captured with a high angle annular dark field (HADDF) detector. A beam current of 45 pA, beam convergence of 30 mrad, and camera length of 115 mm are used for image acquisition. The low magnification STEM images of the aligned and misaligned triangular domains are shown in Figure S1 a-b respectively. Mechanical cracks are observed in both cases. The HRSTEM images shown in the Figure 1g, h are taken from the labeled regions. In addition to the grain boundary in the monolayer MoS_2, we extend this characterization to bilayer aligned MoS_2 (Figure S1 c). Similar to the monolayer case, there is no grain boundary observed when two aligned bilayer MoS_2 domains merge (Figure S1 d).

[1] jrobinson@psu.edu

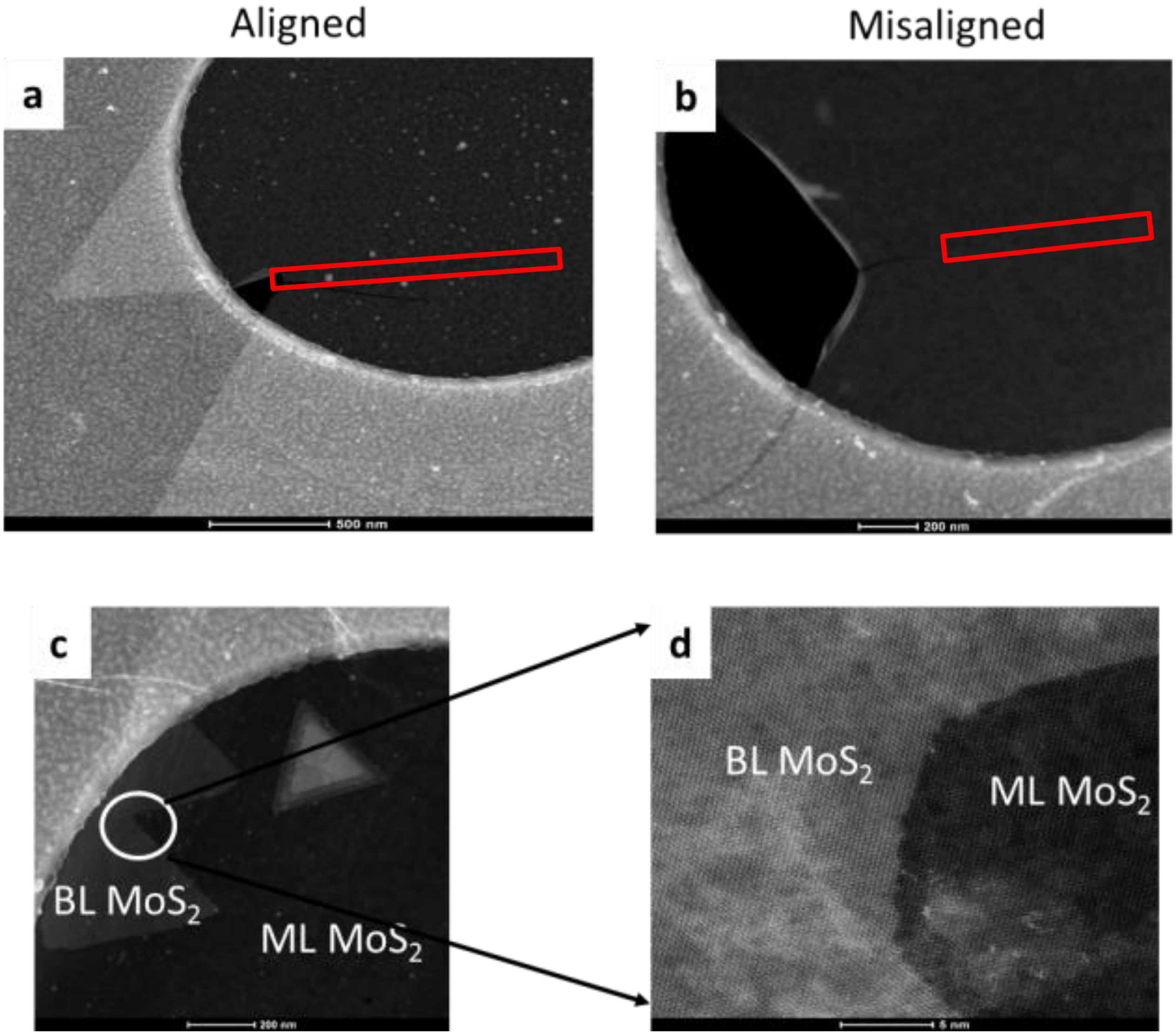

Figure S1- The HRSTEM image of the monolayer-bilayer MoS_2. The low-magnification HRSTEM image of two aligned monolayer domains (a) and misaligned monolayer domains (b). The low magnification HRSTEM image of aligned bilayer domains (c), and the atomic-resolved micrograph in the grain boundary region (d).

Raman characterization of grain boundaries between aligned domains

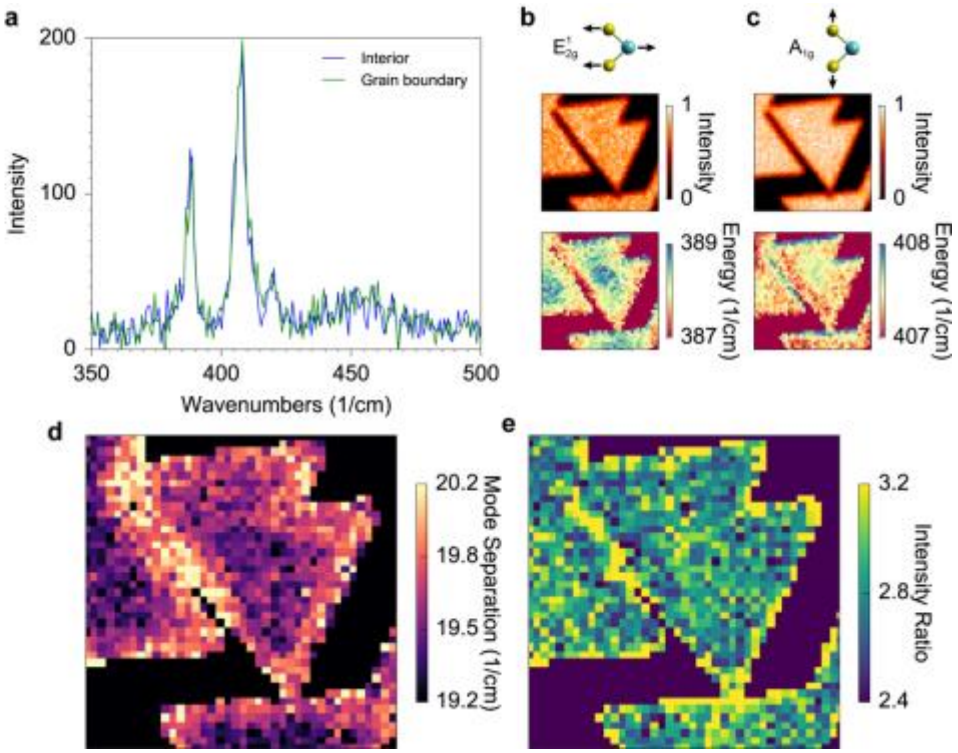

Figure S2 – Hyperspectral μRaman characterization of the multi-flake aggregate of monolayer MoS_2 with the "aligned" grain boundary shown in Fig. 2 of the main text. (a) Sample Raman spectra from the interior (blue curve) and grain boundary (green curve) regions are nearly identical. (b) Maps of the intensity and energy of the lower-energy E_{2g}^1 mode. (c) Similar maps of the intensity and energy of the A_{1g} mode. For both prominent modes, features reminiscent of a grain boundary cannot be observed in the mode intensity or energy. Likewise, neither the spatial map of the separation between the two modes (panel d) nor the intensity ratio of the two modes (panel e) exhibit any features that are indicative of large-scale strain effects within the vicinity of the grain boundary. All of the spatial maps are 12×12 μm^2.

Raman characterization of grain boundaries between misaligned domains

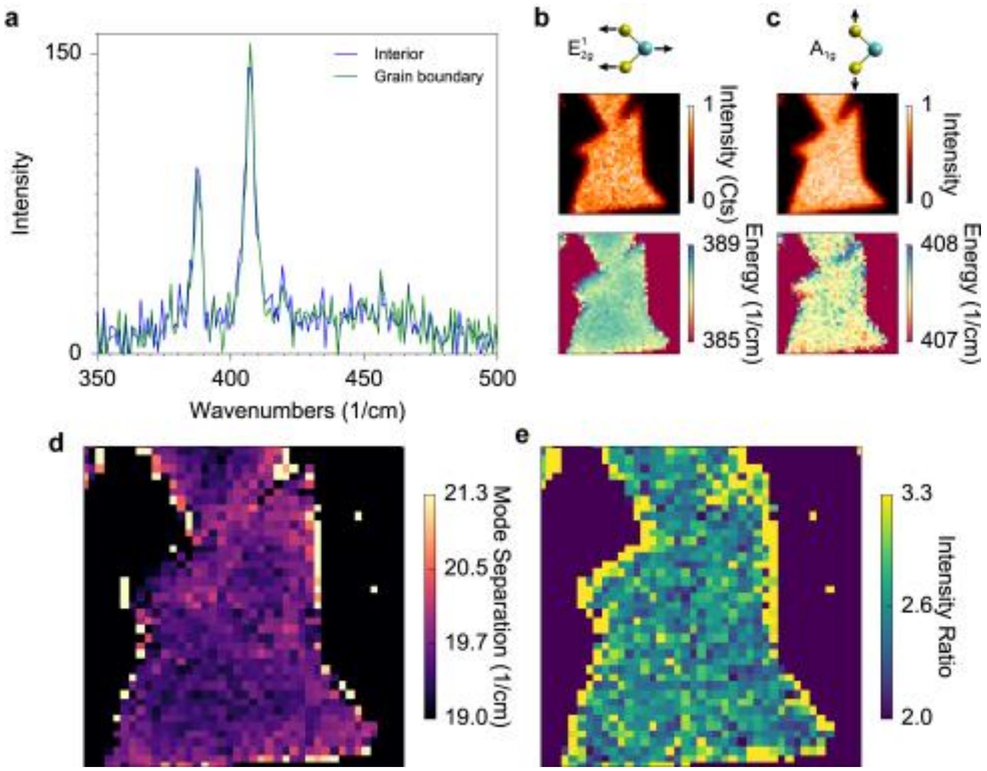

Figure S3 – **Hyperspectral μRaman characterization of the multi-flake aggregate of monolayer MoS_2 with the "misaligned" grain boundary shown in Fig. 1 of the main text.** (a) Sample Raman spectra from the interior (blue curve) and grain boundary (green curve) regions are nearly identical. (b) Maps of the intensity and energy of the lower-energy E_{2g}^1 mode. Despite showing substantially enhanced PL, corresponding features corresponding to the bright grain boundary cannot be observed in intensity or energy of either mode. Likewise, neither the spatial map of the separation between the two modes (panel d) nor the intensity ratio of the two modes (panel e) exhibit any features that are indicative of any significant strain effects within the vicinity of the grain boundary. All of the spatial maps are 12 × 12 μm^2.

Sapphire annealing study

The as received sapphire is cleaned by the following process: acetone sonication for 10 minutes, IPA sonication for 10 minutes, followed by the DI water rinse. After that, the substrate is subsequently soaked in Nanostrip™ at 80˚C for 20 minutes. After the cleaning, the substrate is annealed in the air with the conditions shown in table S1, followed by a second cleaning as described above.

Table S1: The annealing conditions used to tune the sapphire surface properties

1 hour	900 ˚C	1000˚C	1150˚C	1250˚C	1350˚C
8 hour	900˚C	1000˚C	1150˚C	1250˚C	
16 hour	900˚C	1000˚C			
24 hour	900˚C				

Thermal treatment of the sapphire substrate is required to achieve long-range commensurability and epitaxy of MoS_2 on sapphire surfaces.[1,2] Such thermal treatments lead to the formation of atomic steps on sapphire,[3] and the particular annealing conditions can significantly alter the physical size and chemical functionality of the steps, which substantially modifies the quality and yield of monolayer MoS_2. The atomic force microscope (AFM) images of annealed sapphire are shown in Figure S4 (a-l). The nano-steps start to form at 900˚C for 1 hour and the step height is characterized as ~0.2 nm, agreeing with the 1/6 c distance of sapphire.[3,4] The initial steps are zig-zag shape while the step edges become more linear with increased annealing temperature and time. When the annealing temperature/time hit the critical condition (ex: Figure S4 (c, d, h)), the sapphire exhibits a particle-free surface with smooth step edges. The particles and step bunching are observed on sapphire when we keep increasing the annealing temperature and annealing time (ex: Figure S4 (e, i)), indicating the over-etching by the annealing.[3,4]

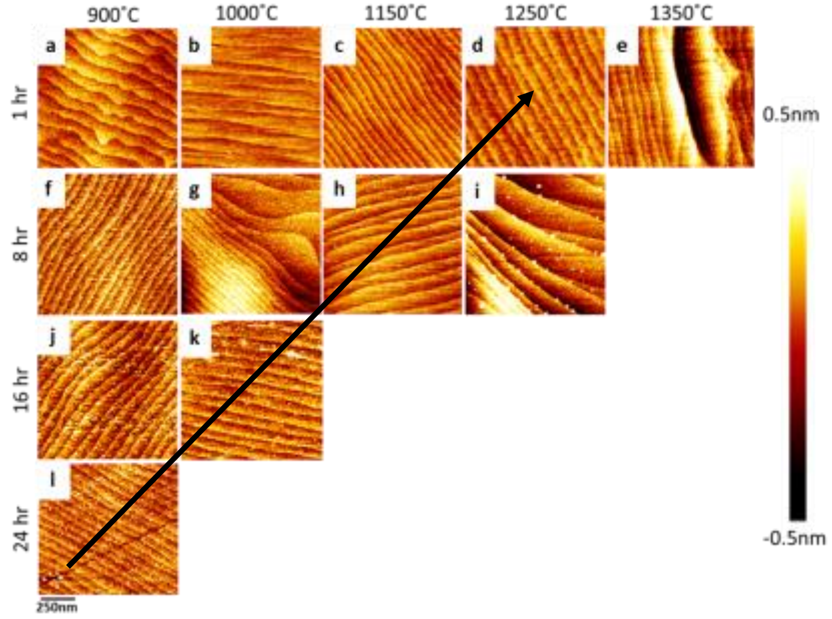

Figure S4- AFM micrographs of thermally treated sapphire substrates. The surface reconstruction is clearly observed when the annealing temperature is > 1000 °C. The optimum surface (smooth terrace edge and particle-free) can be

achieved in the arrow-indicated row (black arrow), indicating the medium strength of annealing (temperature and time) results in the optimum sapphire surface for MoS_2 growth. Strengthening the annealing (increase annealing time and temperature) results in step bunching and particles, and weakening the annealing results in curve steps.

Discontinuous films of monolayer MoS_2 domains are subsequently grown by powder vaporization technique. 2 mg MoO_3 (99.8%, Sigma Aldrich) is put in an alumina crucible located in the center of a hot wall furnace. 200 mg of S powder (99.995%, Alfa Aesar) is put in a quartz crucible located ~12 inches upstream of the MoO_3 crucible. After 5 min of flowing 100 sccm Ar at 710 Torr, the furnace is elevated to 550 ˚C for 2 min as a nucleation step, followed by dwelling at 800 ˚C for 15 min for the growth. The sulfur powder is heated by an individual heat tape wrapped outside the furnace, and the temperature is 130 ˚C. The as grown MoS_2 on sapphire is characterized by AFM, scanning electron microscope (SEM) and Raman spectroscopy (Figure S5 (a-c), respectively). The AFM image shows triangular MoS_2 domain, and the thickness is ~6.7 Å, agreeing with previous reports for monolayers.[5–7] The low magnification SEM image shows there is no alignment. Three peaks: E_{2g} (385.1 cm^{-1}), A_{1g} (402.9 cm^{-1}) and a tiny sapphire peak (419.1 cm^{-1}) are observed. Interestingly, the A_{1g} is significantly quenched and red shifted compared to some other PV-grown MoS_2 monolayers on SiO_2,[6,8] strongly indicating electron doping and charge transfer to the MoS_2.[9] The photoluminescence (PL) measurement is also carried out at 4.1 mW and in 488nm laser wavelength. The peak position is 1.83 eV, and the PL/A_{1g} ratio is ~1.3. It is clear that PL is quenched and red shifted by 0.5 eV in our case compared to others.[10] The PL change also suggests the electron doping of the MoS_2.[11]

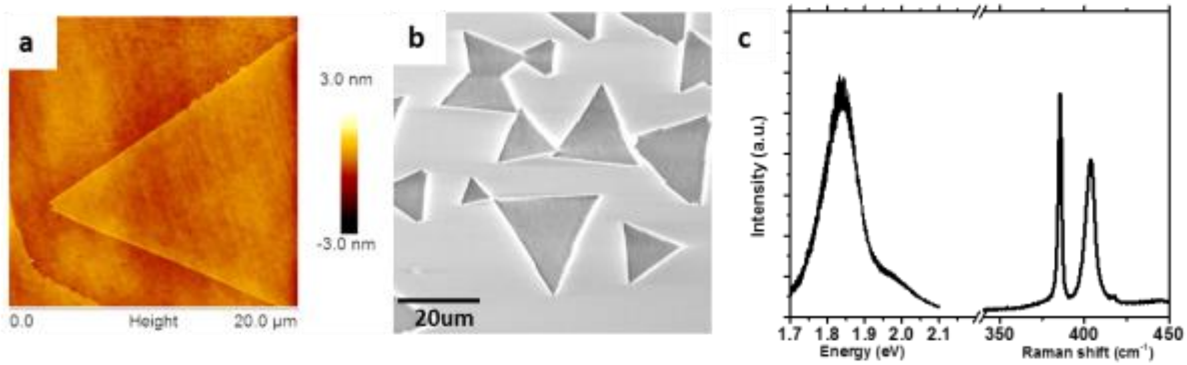

Figure S5- (a). The AFM image of the MoS_2 grown on as-received sapphire, the thickness is ~0.7nm, agreeing with the monolayer MoS_2 reported before. (b). The SEM image of the MoS_2/as-received sapphire, 10-20um triangular domains are observed with random orientation. (c). The PL and Raman spectra of the MoS_2/as-received sapphire. The PL is quenched and 0.6eV red shifted compared the exfoliated samples. The increased A_{1g} and E_{2g} ratio further suggests the electron doping in the MoS_2 monolayer.

The AFM and SEM micrographs of the synthetic MoS_2 monolayers on the sapphire with different thermal treatments are shown in Figure S6 and Figure S7. It is clear that the particle density and alignment varies based on the variation of annealing process.

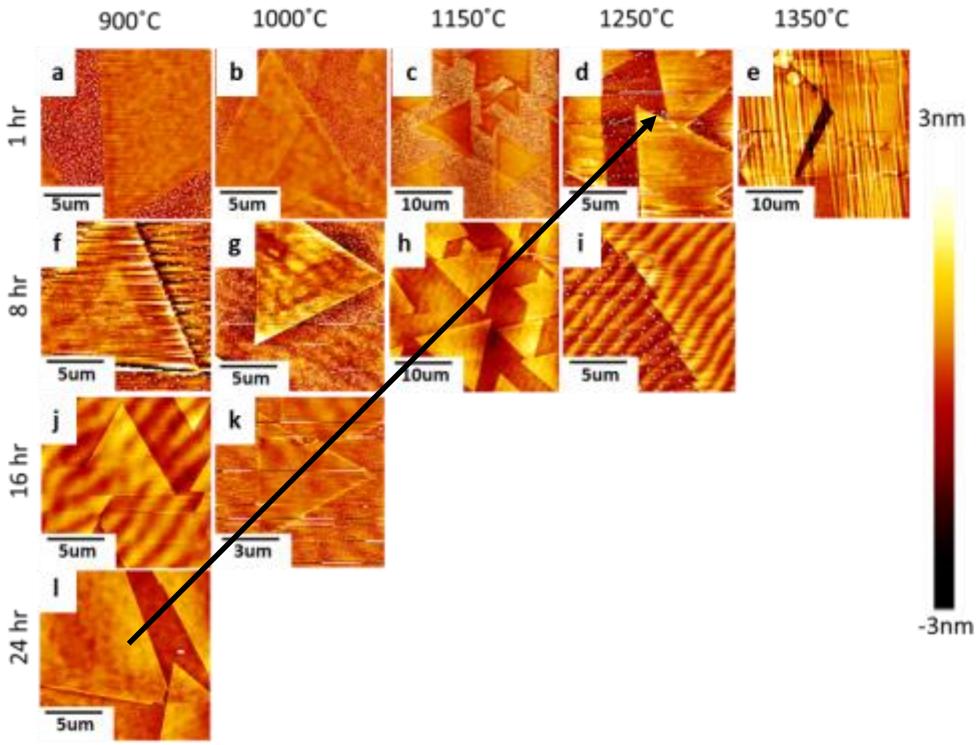

Figure S6- The AFM micrograph of PV-grown MoS_2 on the thermally treated sapphire substrates. The particle-free MoS_2 with good alignment is observed on the substrate treated with optimum conditions. The growth on less-treated sapphire (left side of the arrow) and over-treated (right side of the arrow) exhibits clear nanoparticles with reduced alignment.

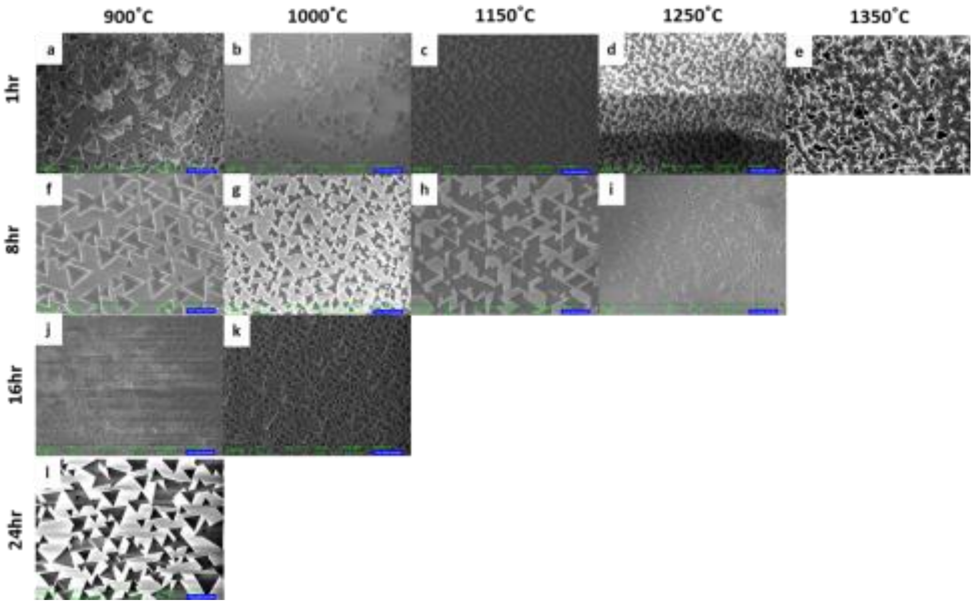

Figure S7- The SEM images of PV-grown MoS_2 monolayer on sapphire that treated by different annealing profile.

For a better comparison, we pick some representative cases and summarized them in Figure S8. As-received sapphire substrates (Fig. S8 (a)), sapphire annealed at 900˚C for 1hr (Fig. S8 (b)), 1150˚C for 8hrs (Fig. S8 (c)), and 1350˚C for 1hr (Fig. S8 (d)) illustrate the evolution in surface topography as a function of annealing conditions. We find no significant morphology changes for sapphire annealed at 900˚C for 1hr compared to the as-received sapphire. In contrast, sapphire annealed at 1150˚C for 8 hr (Fig. S8 (c)) exhibits significantly smoother step-edges, indicating a surface reconstruction of the sapphire surface.[3] Finally, upon annealing at 1350˚C for 1hr (Fig. S8 (d)), significant step-bunching - where multiple steps merge to form a single larger step - is observed, indicating an "over etching" of the sapphire.[4,12] Subsequently, MoS_2 was grown on the as-received and annealed sapphire substrates (Fig. S8 (e), (f), (g), (h)), where well aligned, particle-free MoS_2 domains are achieved on sapphire annealed at 1150˚C for 8 hr. In contrast, MoS_2 growth on the sapphire substrates annealed under the other two conditions yields lower fractional alignment, as well as "3D" particles. To better understand the role of the substrate annealing process on the MoS_2/substrate interactions, the water contact angle (controlled by the substrate surface energy) before growth is measured, followed by a statistical survey of the MoS_2 inter-domain alignment after the growth reaction for the different annealing conditions. Upon comparison of contact angle on the annealing conditions (Fig. S8 (i)), we clearly find that increased annealing duration reduces the contact angle for all evaluated temperatures up to an optimal time. A summary of the inter-domain alignment (Fig. S8 (j)) reveals that the highest degree of crystalline alignment is observed on the substrate which was annealed at 1250˚C for 1hr, which also exhibits the lowest contact angle. There is an inversely proportional trend between contact angle (surface energy) and alignment percentage and domain size. As the surface energy (contact angle) increases (decreases), the sapphire surface becomes more reactive due to a higher density of ordered Al- and O- dangling bonds on the surface. This ultimately leads to enhanced nucleation on the sapphire surface and a reduced energy barrier to achieve long-range registry of the MoS_2 atoms with the Al_2O_3.[2,13] Interestingly, at the lowest evaluated annealing temperature (900˚C), the alignment is more dominated by the annealing time rather than contact angle (Fig. S8 (i, j)), suggesting that under certain conditions one is able to achieve similar surface chemistries required for alignment, while maintaining a relatively low surface energy (high contact angle). In order to understand the surface chemistry of the sapphire, angle resolved XPS (ARXPS) was used. (Figure S9) A Phi Versa Probe II is used for this analysis, with a passing energy of 23.5 eV and a step size of 0.1 eV. All of the samples are integrated at the same dwell time (200 ms) at each step. There is no noticeable beam damage to the sample. The high-resolution spectra for Al at various temperatures did not change. There was no noticeable change as we went down to 30^0 which would result in observing the top 1 nm of the sample surface. It can be noted that the intensity went down as the total probe depth decreased to 1 nm. There is no change in the overall peak structure, indicating that the surface has the same composition similar to the bulk of the substrate. This suggests that the large, systematic changes in the surface energy with annealing (Figure S8 (i, j)) may be the

result of the removal of defective (or even amorphous) Al_2O_3 at the surface generated by the polishing process during wafer manufacturing.

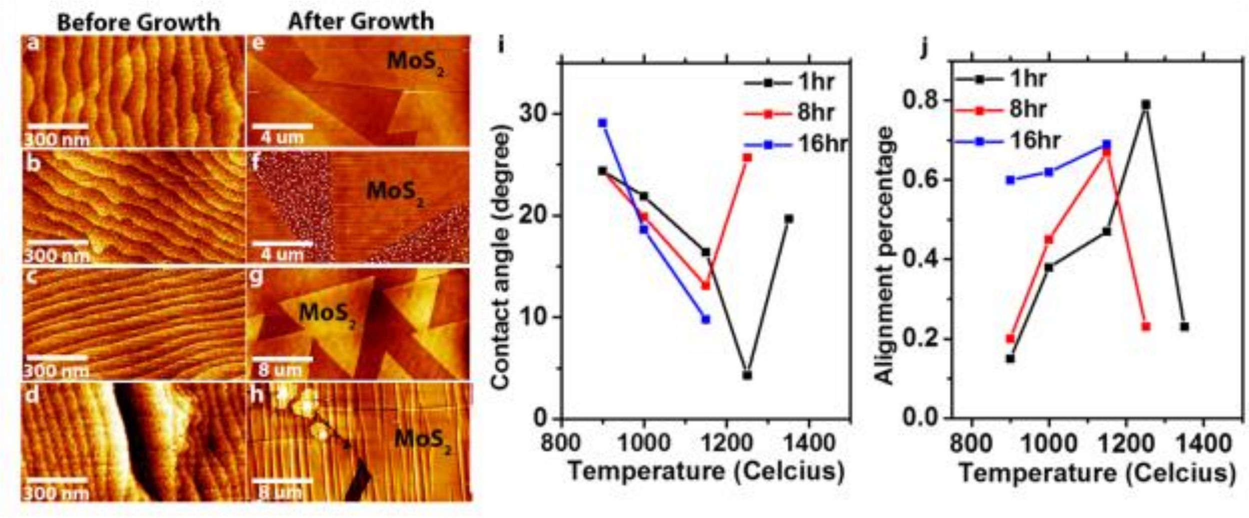

Figure S8- The role of the morphology and surface energy of inter-domain alignment of monolayer MoS_2 grown on sapphire. (a-d) the AFM micrographs of the as-received sapphire (a), sapphire annealed at 900°C for 1 hr (b), sapphire annealed at 1150°C for 8 hr (c) and sapphire annealed at 1350°C for 1 hr (d). It is clear that there is not a significant surface morphology change after annealing at 900°C for 1 hr. In contrast, smoother steps are observed on the 1150°C 8 hr annealed sapphire, indicating effective surface reconstruction of the sapphire after the thermal treatments. Step bunching is observed on the sapphire that was annealed at 1350˚C for 1 hr, indicating the over-etching of the sapphire. (e-h) the AFM micrographs of the monolayer MoS_2 grown on the sapphire substrates that were annealed under the corresponding conditions (by row) specified in (a-d). While particle-free, triangular MoS_2 is grown on the as-received sapphire (e), it is misaligned. The well-aligned and particle free MoS_2 is grown on the 1150°C 8 hour annealed sapphire (g), and imperfect alignment and particles are found in the other two cases (f and h). (i-j) the dependence of the contact angle of the sapphire surface (i) and the alignment fraction of MoS_2 (j) on annealing temperatures for various annealing times. The contact angle can be decreased by strengthening the annealing (i.e., increasing temperature and time) to a minimum value, which yields the maximum alignment fraction.

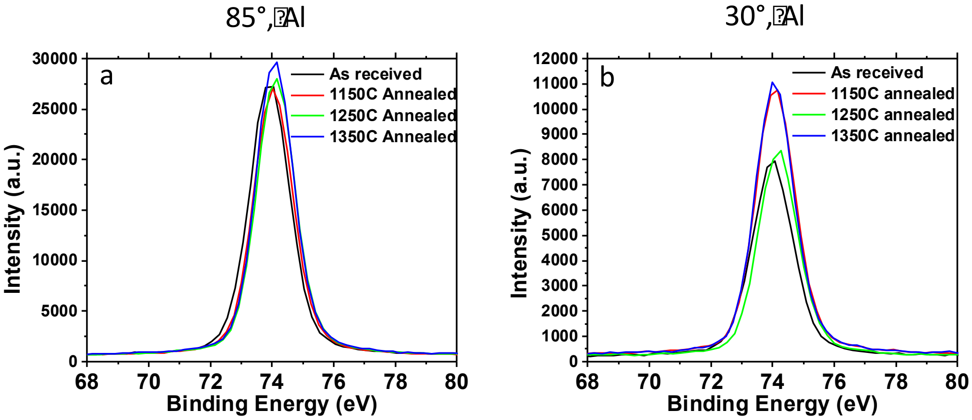

Figure S9 – The ARXPS spectra of sapphire after thermal treatment. The ARXPS spectra in Al 2p range at 85° (a) and at 30° (b). It is clear that there is no significant peak shift and peak shape change after thermal treatment. Note that the peak intensity is higher when the detection angle is at 85° than it is at 30°, which is due to the probe thickness is higher at 85°.

Sapphire surface termination

The surface termination of sapphire can be modeled and determined by visualizing the height profile of the sapphire surface.[14] To understand the surface termination of our sapphire, we conducted a careful AFM characterization on our as-received c-sapphire, annealed sapphire (1150 ˚C, 8 hours) and as received r-sapphire, respectively (Figure S10 (a-c)). The height profile is extracted in Figure S10 (d-f) as black line, and the height profile of the reference[14] is drawn in red. It is clear that the height profiles of the as-received c-sapphire, annealed sapphire and r-sapphire match the referenced height profile of Al-terminated sapphire, Al-O terminated sapphire and O-terminated sapphire, respectively, indicating the corresponding surface terminations of our sapphire substrates. Interestingly, the step width of our r-sapphire is 3× larger than that of the referenced O-terminated sapphire, which can be due to the larger O-O distance on the r-sapphire surface.[15]

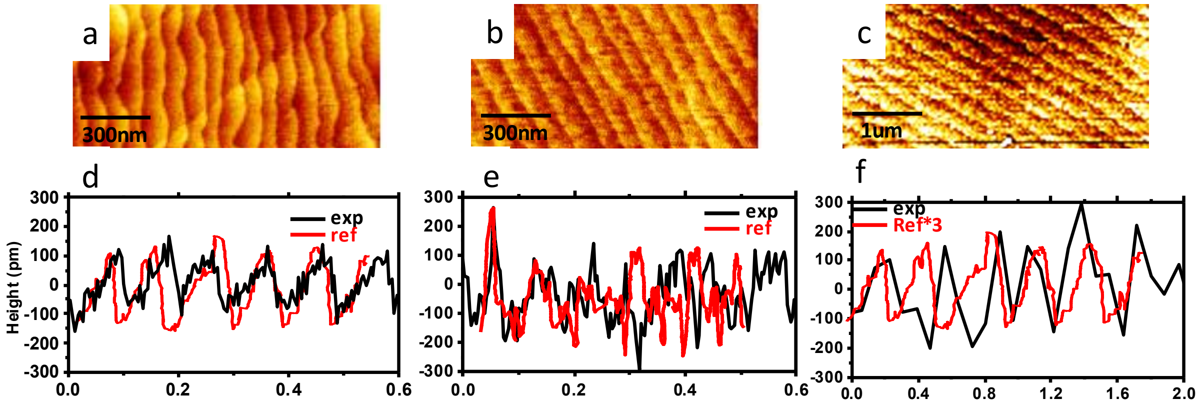

Figure S10- Surface termination of the sapphire substrates. (a-c). the AFM images of as-received c-sapphire, annealed c-sapphire and as-received r-sapphire; (d-f). the corresponding height profile of the sapphire substrates. The black line is the experimental height profile of the sapphire substrates used in this experiment and the red line is the experimental height profile in the reference.

Peak fitting and calculated carrier concentration of MoS_2 on c and r plane sapphire

The PL of monolayer MoS_2 grown on c-sapphire is significantly different from that of monolayer MoS_2 grown on r-sapphire. The PL spectral of MoS_2 on c-sapphire (a) and r-sapphire (b) are shown in Figure S11. After Lorentzian peak fitting, the trion peak (red) and exciton peak (green) is clearly identified at 1.83 eV and 1.87 eV and agrees with the previous reports.[11,16] It is clear that the trion-dominated PL changes to exciton-dominated PL when the growth is on r-sapphire instead of c-sapphire, indicating that less negative charge is transferred to the MoS_2 grown on r-sapphire. From the peak fitting, we are able to estimate the electron concentration of the monolayer MoS_2 on c-sapphire and r-sapphire,[17,18] and the estimated electron concentration is $3.14 \times 10^{12}\ cm^{-2}$ and $9.82 \times 10^{10}\ cm^{-2}$ on c-sapphire and r-sapphire respectively. This value also agrees well with the electron concentration vs. decay time in our measurements and previous reports from Amani *et al.* [19]

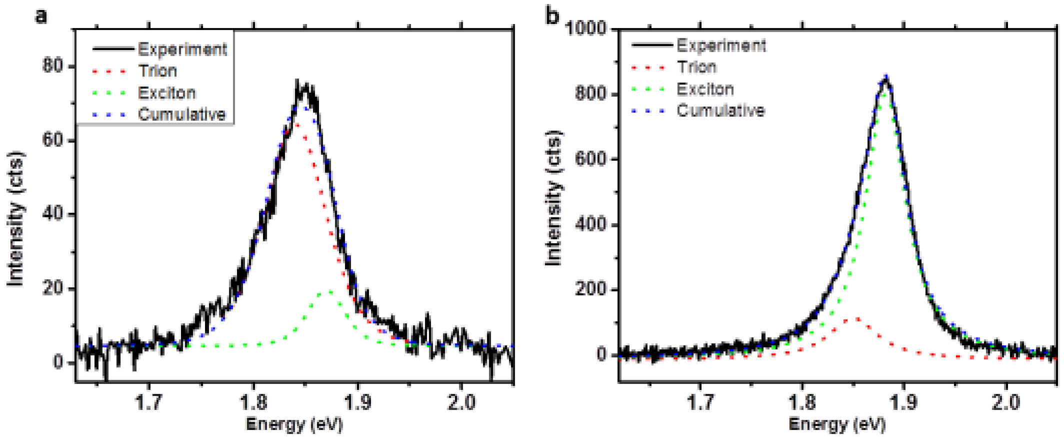

Figure S11- The PL peak fitting of MoS_2 grown on c-sapphire (a) and r-sapphire (b).

Rate equation analysis of power-law scaling and multi-exciton threshold

A simple rate equation can be used to model the multi-exciton effects on the power-law scaling curves reported in Figure 2 of the main text and here in the SI for edges, grain boundaries and interior regions[20]:

$$\frac{dN}{dt} = -\frac{1}{\tau_{exc}}N - k_{ee}N^2$$

where N is the density of excitons, t is the time after pulsed excitation, τ_{exc} is the excited state lifetime due to radiative and non-radiative recombination in absence of multi-exciton effects, and k_{ee} is the exciton-exciton annihilation rate. This equation can be solved analytically for:

$$N(t) = \frac{N_0 k_{exc}}{e^{-t \cdot k_{exc}}(k_{exc} + k_{ee}N_0) - k_{ee}N_0}$$

where N_0 is the initial exciton density created by an optical excitation pulse, and $k_{exc} = 1/\tau_{exc}$ is the excited state relaxation rate (in absence of multi-exciton effects). From this solution, the time-integrated emission intensity for different initial exciton densities (i.e., N_0) is can be calculated by numerically integrating $N(t)$:

$$I(N_0) = \int_0^{\infty} N(t; N(0) = N_0) dt$$

Figure S11 shows the dependence of the time-integrated emission on excitation density for two different excited state lifetimes. For these numerical calculations, we used an exciton-exciton annihilation rate of $k_{ee} = 4.3\ cm^2/s$ that was determined in previous studies[21] and calculated N_0 assuming 5% absorption at an excitation energy of 2.40 eV. The excited state lifetimes used were extracted from the PL decay transients in Figure 3 by performing a fitting a convolution of $N(t)$ with the instrument response function of our experimental setup to enable a rough comparison to the data of Figure 2. A decrease in the excited state lifetime by ~100×, which is comparable to the differences between the r-sapphire and c-sapphire substrates in the main text, results in a power-law scaling that is largely linear over these excitation densities. However, with a longer excited-state lifetime, the transition from a linear to sublinear power-dependence occurs at a much lower excitation density. These trends qualitatively reproduce what is observed in the main text for the c-sapphire and r-sapphire substrates and further support the hypothesis that non-radiative rate suppression plays a significant role in the enhancement of the photoluminescence of MoS_2 on sapphire and at the edges and misaligned grain boundary regions.

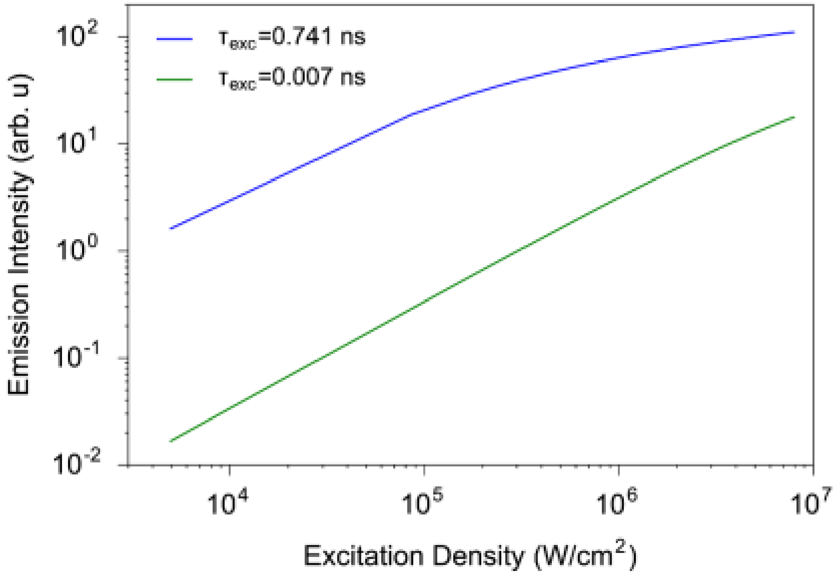

Figure S12 – Dependence of the excitation density threshold that marks the onset of multi-exciton dominated recombination.

Field effect transistors (FET) device fabrication

Following MoS_2 growth on sapphire substrates, an array of Ti/Au (10/90 nm) alignment/fiduciary are deposited by standard optical lithography, e-beam evaporation, and lift-off processes. Large triangular monolayer MoS_2 flakes (40-60 μm side length) are then etched into smaller rectangular channels of varying widths and lengths (dictated by original flake size) to be used as a TLM bar of uniform composition/thickness and defined width. Following electron beam lithography (EBL), the MoS_2 etch is carried out in a Plasma Therm PT-720 Reactive Ion Etch (RIE) tool using a gas chemistry of SF_6/Ar/O_2 (30/10/10 sccm) at 100 W power and 10 mTorr pressure for a total etch time of 20 seconds. Minimal etching of the sapphire substrate is observed. Two subsequent metallization steps are then carried out to fabricate TLM (FET) structures suitable for electrolyte gating in the form of an initial thin source/drain metallization (actually contacting the MoS_2 film) and a secondary large-area thick metallization for easier probing. First, 25 nm Ni source/drain extensions (grey metal fingers in Figure 3a) are formed by EBL, e-beam evaporation, and lift-off processes. Ni is deposited at 0.5 Å/sec at a deposition pressure of $3x10^{-6}$ Torr. The width of these Ni source/drain fingers is 2 μm. Prior to Ni source/drain deposition and after e-beam resist development, samples are O_2/He (150/50 sccm) plasma treated (50 W and 500 mTorr) for 45 seconds in an M4L etch RIE tool in order to chemically remove resist polymer residues and thereby improve the metal/MoS_2 interface. Finally, 15/165 nm Ti/Au interconnects and pads are deposited to ensure ease of probing. In addition to thick pads and leads, a “side-gate” structure adjacent to the TLM structure is also written in this same EBL step; this side-gate is electrically isolated from the MoS_2 channel and source/drain contacts, and is used to apply a voltage to the electrolyte with a third-terminal probe which is discussed in more detail in the electrolyte measurement section. This device geometry is chosen for several reasons. First, it provides a constant gate-MoS_2 distance (~ 6 μm) and electric field which allows for consistent measurements (i.e. sweep rate) between devices. Secondly, it is much easier to make electrical contact with a probe-metal pad configuration than it is to consistently insert a probe into a thin electrolyte directly above a device at a constant height.[22]

Field effect transistors (FET) device fabrication

Following MoS_2 growth on sapphire substrates, an array of Ti/Au (10/90 nm) alignment/fiduciary are deposited by standard optical lithography, e-beam evaporation, and lift-off processes. Large triangular monolayer MoS_2 flakes (40-60 μm side length) are then etched into smaller rectangular channels of varying widths and lengths (dictated by original flake size) to be used as a TLM bar of uniform composition/thickness and defined width. Following electron beam lithography (EBL), the MoS_2 etch is carried out in a Plasma Therm PT-720 Reactive Ion Etch (RIE) tool using a gas chemistry of SF_6/Ar/O_2 (30/10/10 sccm) at 100 W power and 10 mTorr pressure for a total etch time of 20 seconds. Minimal etching of the sapphire substrate is observed. Two subsequent metallization steps are then carried out to fabricate TLM FET structures suitable for electrolyte gating in the form of an initial thin source/drain metallization (i.e., the contact metals that actually touch the MoS_2) and a secondary, large-area thick metallization to provide robust contacts that can withstand repeated probing. First, 25 nm Ni source/drain extensions (grey metal fingers in Figure 3a) are formed by EBL, e-beam evaporation, and lift-off processes. Ni is deposited at 0.5 Å/sec at a deposition pressure of $3x10^{-6}$ Torr. The width of these Ni source/drain fingers is 2 μm. Prior to Ni source/drain deposition and after e-beam resist development,

samples are O_2/He (150/50 sccm) plasma treated (50 W and 500 mTorr) for 45 seconds in an M4L etch RIE tool in order to chemically remove resist polymer residues and thereby improve the metal/MoS_2 interface. Finally, 15/165 nm Ti/Au interconnects and pads are deposited to ensure ease of probing. In addition to thick pads and leads, a "side-gate" structure adjacent to the TLM structure is also written in this same EBL step; this side-gate is electrically isolated from the MoS_2 channel and source/drain contacts, and is used to apply a voltage to the electrolyte with a third-terminal probe which is discussed in more detail in the electrolyte measurement section. This device geometry is chosen for several reasons. First, it provides a constant gate-MoS_2 distance (~ 6 μm) and electric field which allows for consistent measurements (i.e. sweep rate) between devices. Secondly, it is much easier to make electrical contact with a probe-metal pad configuration than it is to consistently insert a probe into a thin electrolyte directly above a device at a constant height.[22]

Electrolyte gating application

A solid polymer electrolyte (PEO:$CsClO_4$) is used for ionic gating. The preparation of the polymer electrolyte is similar to previously published procedures[23] with the exception that the electrolyte is prepared and deposited in an argon-filled glovebox where the concentrations of H_2O and O_2 are maintained to be < 0.1 part-per-million (ppm). Poly(ethylene-oxide) (PEO) (molecular weight 95,000 g/mol, Polymer Standards Service) and anhydrous $CsClO_4$ (99.9%, Sigma-Aldrich) are dissolved in anhydrous acetonitrile (Sigma-Aldrich) with an ether oxygen to Cs molar ratio of 76:1 to make a 1 wt% solution. The solid polymer electrolyte is deposited on the sample by drop-casting 25 μL onto the ∼1 x 1 cm^2 sample. After a 15 minute wait time to allow the majority of the solvent to evaporate, the sample is annealed on a hotplate at 80 °C for 3 mins to drive off remaining solvent. The sample is then transferred from the glovebox to the probe station through an Ar-filled load lock. The entire process of electrolyte preparation, deposition, transfer to the probe station, and measurement are completed under an inert gas environment with no sample exposure to ambient. Electrical measurements are performed on a Lake Shore cryogenic vacuum probe station (CRX-VF) under ∼ 10^{-6} Torr at room temperature using a Keysight B1500A semiconductor parameter analyzer.

Film tearing and delamination

Both MoS_2/c-sapphire and MoS_2/r-sapphire samples are processed with the identical procedure as follows: both samples are dip-coated in Surpass 4000 adhesion promoter prior to spin-coating of the e-beam resist ZEP 520A (1:1 ZEP:Anisole) to improve coating over the ~ 100 nm thick metal alignment mark features. This is needed to prevent etching of alignment marks during removal of the sacrificial Au layer (~15 nm) that is thermally evaporated on top of the e-beam resist stack to decrease charging effects during EBL exposure on insulating sapphire substrates. This Au wet etch (TFA Au Etchant) is performed after EBL exposure and prior to resist development. Following 45 sec Surpass dip, samples are then placed in DI water, and N_2 dried. After the process, we find that MoS_2/c-sapphire sample displays partially torn channels while all MoS_2/r-sapphire channels remain intact. This discrepancy in 'adhesion' to the substrate could be due to the increased chemical bonding with the substrate in the case of r-sapphire. Meanwhile, the MoS_2/r-sapphire samples exhibit micro-cracking within the MoS_2 monolayer film which is observed predominantly for channel lengths of 1 μm and smaller. These micro-cracks can be seen running parallel and directly adjacent to the source/drain fingers of the TLM structure. This micro-cracking is observed only after deposition and lift-off of Ni contacts. This is also observed for

MoS_2 on c-plane sapphire but with less frequency and for mainly 0.5 μm channels and smaller. This minor discrepancy could also have to do with the difference in degree of chemical bonding between MoS_2 and the underlying substrate for r-plane vs c-plane, which could impact the ability of a monolayer film to respond to and accommodate external stresses. In qualitative terms, the MoS_2 monolayers on r-sapphire devices are more constrained by the substrate, which could lead to critical mechanical failure possibly experienced during metal deposition from a combination of strain and thermal cycling. Nevertheless, this tendency to fracture for both cases could have important implications for channel length scaling on sapphire substrates and is worth further investigation on how to potentially mitigate this issue in the future.

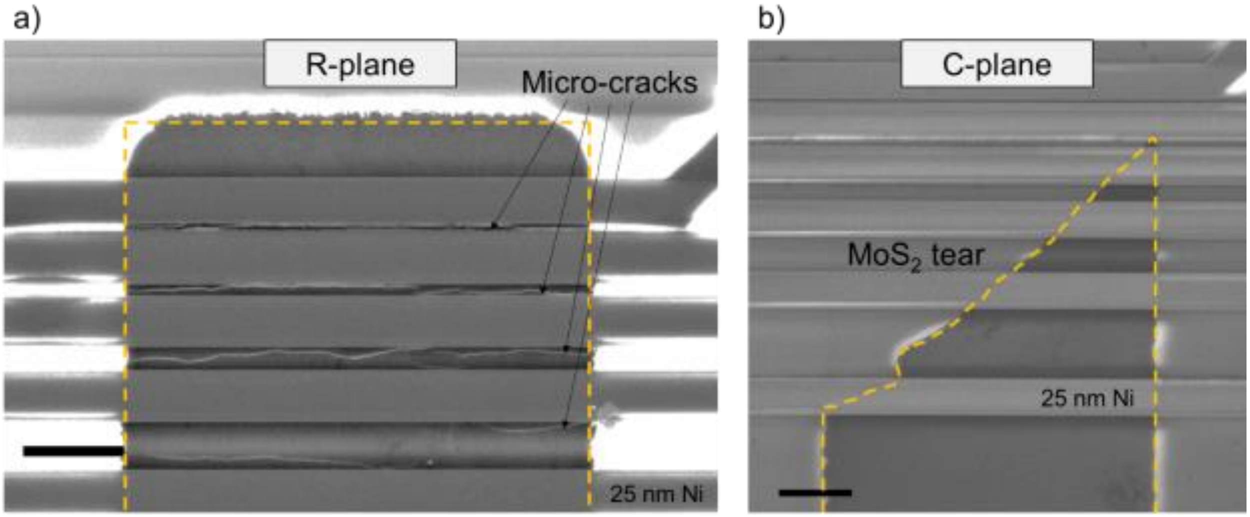

Figure S13- Processing impacts on MoS_2. (a) An SEM image of a TLM structure on MoS_2/r-sapphire showing the micro-cracking in the monolayer MoS_2 film between Ni (25 nm) contacts. In this particular device, cracks can be seen for devices with channel lengths of 0.125, 0.25, 0.5, and 1 μm, which is largely typical for MoS_2/r-sapphire. Cracking is normally observed for channel lengths < 1 μm. (b) An SEM image of a TLM structure on MoS_2/c-sapphire showing the tearing and delamination of monolayer film from the substrate encountered during device fabrication, observed on most TLM devices on c-sapphire. In addition, cracking was also observed in MoS_2/c-sapphire devices but to a lesser extent (not shown). No tearing or delamination was observed for MoS_2/r-sapphire, except for 1 out of 16 TLM structures. Scale bars are 2 μm.

Transfer Length Measurement (TLM)

Prior to doing any gated measurements (transfer, output characteristics) on a particular TLM structure corresponding to an individual flake, two-terminal IV curves were measured on all devices of varying

channel lengths with a voltage range of -100 - 100mV (V_g = 0V) (Figure S13)

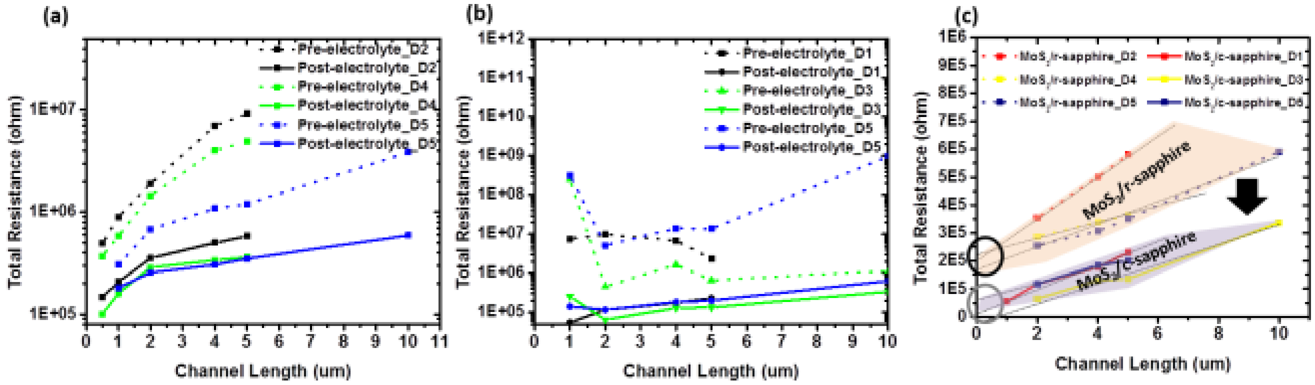

Figure S14-Contact resistance and sheet resistance extraction for MoS_2/r-sapphire and MoS_2/c-sapphire devices by transfer length method (TLM). (a) Plots of total device resistance normalized by channel width, W, (log scale) vs channel length for MoS_2/r-sapphire for 3 separate TLM structures both pre (in-air) and post electrolyte deposition, showing a reduction in the total resistance of each device following electrolyte application. Total resistance is determined by 2 terminal IV measurements in both cases, in-air (ambient) and after electrolyte application. Electrolyte measurements are carried out in a Lakeshore probe station under vacuum (10^{-6} Torr) at room temperature. No gate bias is applied during IV measurements taken post electrolyte application. (b) Similar TLM plots for MoS_2/c-sapphire for 3 separate TLM structures both pre (in-air) and post electrolyte deposition, showing a similar trend observed for MoS_2/c-sapphire. It is important to note that total resistance (y-axis) is plotted in log-scale for (a-b) in order to more clearly display the change in magnitude as a result of electrolyte deposition, which is consistent across every TLM structure and device on both samples. Resistance data from devices with channel lengths below 0.5 µm (1 µm) for MoS_2/r-sapphire (MoS_2/c-sapphire) were not included due to extremely high resistance values or negligible current (open-circuit), most likely from channel cracking and tearing discussed in Figure S12. The reduction in total resistance is due to a combination of both decreased sheet resistance (linear slope) and contact resistance (linear y-intercept) from ionic electrostatic doping. In fact, TLM data is unreliable prior to electrolyte deposition; there is more scatter in the data, and linear extrapolation of the data gives a negative y-intercept and hence a negative contact resistance. (c) TLM plots for the same device sets from (a-b) from both MoS_2/c-sapphire and MoS_2/r-sapphire, plotted in linear scale, showing the lower total resistances for MoS_2/c-sapphire compared with MoS_2/r-sapphire. Contact resistance, sheet resistance, transfer length, and specific contact resistivity values are tabulated in Table S2 below.

	R-sapphire (post electrolyte deposition; V_g = 0V)				C-sapphire (post electrolyte deposition; V_g = 0V)			
TLM	Device2	Device4	Device5	**Average**	Device1	Device 3*	Device5	**Average**
R_c (kΩ·µm)	102.0	119.1	71.7	**97.6**	10.7	-5.13	30.9	**20.8**
R_{sh} (kΩ/□)	75.0	25.0	44.1	**48.0**	41.3	34.2	17.1	**29.2**
L_t (µm)	1.36	4.76	1.59	**2.57**	0.227	-	1.33	**0.779**
ρ_c (Ω·cm²)	1.39E-03	5.67E-03	1.14E-03	**2.73E-03**	2.42E-05	-	4.12E-04	**2.18E-4**
Channel lengths used for calculation	2, 4, 5	2, 4, 5	1, 2, 4, 5, 10		1, 2, 4, 5	2, 4, 10	2, 4, 5	
R^2 linear fit parameter	0.9995	0.9997	0.9894		0.9822	0.9998	0.9774	

Table S2-Summary of TLM data for MoS_2/r-sapphire and MoS_2/c-sapphire devices. (a) Contact resistance (R_c), sheet resistance (R_{sh}), transfer length (L_t), and specific contact resistivity (p_c) for each TLM structure/device is shown. Also included in this table are the exact channel lengths used for linear fitting as well as the linear fit coefficient (R^2). *Denotes linear trendline fit with a negative y-intercept, and hence negative contact resistance, which is not a realistic.

Electrolyte gating measurements

All transfer curves (I_d-V_g) shown are taken by sweeping V_g from +3 to -4 V. Prior to collecting each transfer curve, a constant gate bias of $V_g = 3$ V is applied for 5 min to allow the ions in the electrolyte/MoS_2 system to reach equilibrium. A 5 min hold time prior to initiating transfer curve measurement is determined by monitoring I_d under constant gate bias ($V_g = 3$ V) and small drain bias over time; when I_d is stabilized, this indicates equilibrium has been established. Because ion transport is slow compared to electrons/holes, sufficient time must be provided for the ions to respond to the changing gate bias (i.e., slow sweep rates are required to ensure repeatability and reduce hysteresis). All transfer curves reported in this manuscript, unless otherwise stated, are initially swept from +3 to -4 V with voltage steps of 150 mV and a dwell time between voltage steps of 30 sec under a drain bias of 500 mV, corresponding to a sweep rate of 5 mV/s. For each sample, devices were first measured using multiple sweep rates to determine the sweep rate that yields the most reproducible results with the smallest hysteresis. A sweep rate of 5 mV/s was determined as the optimal sweep rate because further decreasing the rate did not change the transfer measurement to an appreciate extent. To ensure a fair device-device and sample-sample comparison, all transfer curves are taken with the same sweep direction and sweep conditions.

Field-effect mobility calculations

Field-effect mobility (μ_{FE}) was calculated using the equation $\mu = \frac{g_m \cdot L_{ch}}{W_{ch} \cdot C_{EDL} \cdot V_{ds}}$. In this case, g_m is the transconductance of the device which is the slope of the transfer curve (I_d-V_g) in the linear region (when plotted in linear scale). L_{ch} and W_{ch} are the length and width of the device channel, respectively. $V_{ds} = 500$ mV is the drain bias that was used for all transfer curve measurements and subsequent calculations of V_{th} and SS reported in the main text. C_{EDL} in this case is the capacitance of the electrolyte double layer that is formed at the interface. For PEO:$CsClO_4$ on monolayer MoS_2, a $C_{EDL} = 1$ $\mu F/cm^2$ was used.

References

1. Chen, L. *et al.* Step-Edge-Guided Nucleation and Growth of Aligned WSe_2 on Sapphire via a Layer-over-Layer Growth Mode. *ACS Nano* **9,** 8368–8375 (2015).
2. Dumcenco, D. *et al.* Large-Area Epitaxial Monolayer MoS $_2$. *ACS Nano* **9,** 4611–4620 (2015).
3. Yoshimoto, M. *et al.* Atomic-scale formation of ultrasmooth surfaces on sapphire substrates for high-quality thin-film fabrication. *Appl. Phys. Lett.* **67,** 2615 (1995).
4. Ribic, P. R. & Bratina, G. Behavior of the (0 0 0 1) surface of sapphire upon high-temperature annealing. *Surf. Sci.* **601,** 44–49 (2007).
5. Lee, Y.-H. *et al.* Synthesis of large-area MoS2 atomic layers with chemical vapor deposition. *Adv. Mater.* **24,** 2320–5 (2012).
6. Najmaei, S. *et al.* Vapour phase growth and grain boundary structure of molybdenum disulphide

atomic layers. *Nat. Mater.* **12,** 754–9 (2013).

7. van der Zande, A. M. *et al.* Grains and grain boundaries in highly crystalline monolayer molybdenum disulphide. *Nat. Mater.* **12,** 554–61 (2013).

8. Li, H. *et al.* From Bulk to Monolayer MoS2: Evolution of Raman Scattering. *Adv. Funct. Mater.* **22,** 1385–1390 (2012).

9. Chakraborty, B. *et al.* Symmetry-dependent phonon renormalization in monolayer MoS 2 transistor. *Phys. Rev. B - Condens. Matter Mater. Phys.* **85,** 2–5 (2012).

10. Splendiani, A. *et al.* Emerging photoluminescence in monolayer MoS2. *Nano Lett.* **10,** 1271–5 (2010).

11. Mouri, S., Miyauchi, Y. & Matsuda, K. Tunable photoluminescence of monolayer MoS2 via chemical doping. *Nano Lett.* **13,** 5944–5948 (2013).

12. Lucht, M., Lerche, M., Wille, H., Shvyd, Y. V & R, H. D. Precise Measurement of the Lattice Parameters of Sapphire in the Temperature Range 4 . 5 K - 250 K Using the M ossbauer Wavelength Standard 1 Introduction 2 Method. *J. Appl. Crystallogr.* **36,** 1075–1081 (2003).

13. Ji, Q. *et al.* Unravelling Orientation Distribution and Merging Behavior of Monolayer MoS2 Domains on Sapphire. *Nano Lett.* **15,** 198–205 (2015).

14. Wang, R. *et al.* Atomic Step Formation on Sapphire Surface in Ultra-precision Manufacturing. *Sci. Rep.* **6,** 29964 (2016).

15. Pishchik, V., Lytvynov, L. a. & Dobrovinskaya, E. R. *Sapphire*. (2009). doi:10.1007/978-0-387-85695-7

16. Li, Y. *et al.* Photoluminescence of monolayer MoS_2 on $LaAlO_3$ and $SrTiO_3$ substrates. *Nanoscale* **6,** 15248–15254 (2014).

17. Siviniant, J., Scalbert, D., Kavokin, A. V., Coquillat, D. & Lascaray, J.-P. Chemical equilibrium between excitons, electrons, and negatively charged excitons in semiconductor quantum wells. *Phys. Rev. B* **59,** 1602–1604 (1999).

18. Ron, A. *et al.* Thermodynamics of free trions in mixed type GaAsAlAs quantum wells. *Solid State Commun.* **97,** 741–745 (1996).

19. Amani, M. *et al.* Near-unity photoluminescence quantum yield in MoS2. *Science (80-.).* **350,** 1065–1068 (2015).

20. Yu, Y. *et al.* Fundamental limits of exciton-exciton annihilation for light emission in transition metal dichalcogenide monolayers. *Phys. Rev. B* **93,** 201111 (2016).

21. Sun, D. *et al.* Observation of Rapid Exciton–Exciton Annihilation in Monolayer Molybdenum Disulfide. *Nano Lett.* **14,** 5625–5629 (2014).

22. Bersch, B. M. *et al.* Selective-area growth and controlled substrate coupling of transition metal dichalcogenides. *2D Mater.* **4,** 25083 (2017).

23. Xu, H., Fathipour, S., Kinder, E. W., Seabaugh, A. C. & Fullerton-Shirey, S. K. Reconfigurable Ion Gating of 2H-$MoTe_2$ Field-Effect Transistors Using Poly(ethylene oxide)-$CsClO_4$ Solid Polymer Electrolyte. *ACS Nano* **9,** 4900–4910 (2015).
